# Supplementary material for: Five-Decade Prevalence of Delirium in Pneumonia, Risk Factors, and Associated Mortality: A Systematic Review and Meta-Analysis
Source: medRxiv. 2025 Jun 1:2025.06.01.25328725. Preprint. [Version 1] doi: 10.1101/2025.06.01.25328725 (PMC12148281; doi:10.1101/2025.06.01.25328725)
Supplement: Supplement 1 [file media-1.pdf]

Prevalence of Delirium in Pneumonia and its Risk Factors: Systematic Review and Meta-Analysis

Supplementary Online Content

Erika L. Juarez-Martinez[1], Aida Araia[1], Dillan Prasad[1], Shreya Dhar[1], Khizar Nandoliya[1], Ian G. Sherrington[1], Catherine Zhao[1], Annie Wescott[2], Chiagozie I. Pickens[3], Richard G. Wunderink[3], Eyal Y. Kimchi[1]

[1] Ken & Ruth Davee Department of Neurology. Feinberg School of Medicine. Northwestern University. Chicago, Illinois, 60611

[2] Galter Health Sciences Library & Learning Center, Feinberg School of Medicine, Northwestern University, Chicago, IL, 60611

[3] Division of Pulmonary and Critical Care, Department of Medicine. Feinberg School of Medicine. Northwestern University. Chicago, Illinois, 60611

Methods. Data extraction and management

Table S1. Search Strategies across databases
Table S2 Number of papers retrieved from the databases searched
Table S3. Sensitivity Analysis Grading Criteria. JBI Critical Appraisal Checklist for Analytical Cross-Sectional Studies
Table S4. Included studies and their characteristics (*part 1*)
Table S5. Included studies and their characteristics *(part 2)*
Table S6. Relationship of delirium to Baseline characteristics, Comorbidities, measures of Pneumonia Severity and Acute Care, Mortality, and Length of Clinical Care in studies at low risk of bias.

Figure S1. Traffic light plot for risk of bias in individual studies
Figure S2. Funnel plots for all the included studies suggests the presence of publication bias
Figure S3. Forest plot by risk of bias assessment.
Figure S4. Forest plot by Delirium assessment method
Figure S5. Forest plot by Hospital Setting
Figure S6. Forest plot by Pneumonia Infection Origin
Figure S7. Forest plot by Microbiological etiology
Figure S8. Subgroup analysis in studies at low risk of bias
Figure S9. Forest plots per subgroup analysis in studies at low risk of bias
Figure S10. Older age is a predisposing factor for delirium.
Figure S11. Forest plots by demographics as predisposing factors of delirium
Figure S12. Forest plots by comorbidities as predisposing factors of delirium
Figure S13. Forest plots by demographics as predisposing factors of delirium in studies at low risk of bias
Figure S14. Forest plots by comorbidities as predisposing factors of delirium in studies at low risk of bias.

Figure S15. Forest plots by pneumonia-severity factors
Figure S16. Forest plots by pneumonia-severity factors in studies at low risk of bias.
Figure S17. Forest plots of the associations between delirium and clinical course
Figure S18. Forest plots of the associations between delirium and clinical course in studies at low risk of bias
Figure S19. Delirium is associated with significantly increased mortality in patients with pneumonia in studies at low risk of bias.

Supplementary Methods

Data extraction and management

Two out of three authors independently extracted data from studies that fulfilled inclusion criteria (E.K., E.J-M., D.P.). Data was extracted and organized by delirium/encephalopathy status. Any discrepancies were resolved through consensus. The following data were extracted from each study when available:

General study information. Language, study location, start and end date, type of study, and hospital care setting.

Study population. Age, sex, race and ethnicity, comorbidities, Charlson Comorbidity Index,[1] and admission diagnosis.

Pneumonia diagnosis characteristics. Pneumonia diagnostic criteria (i.e., respiratory and infectious signs or symptoms, Chest X-ray, CT scan, chart-based, or International Classification of Disease (ICD) codes), pneumonia origin (i.e., community-acquired (CAP), hospital acquired (HAP), healthcare-associated (HCAP), ventilation associated (VAP)). We integrated VAP and HAP to HCAP given the low numbers of studies that reported these origins. Microbiological etiology (i.e., bacterial, COVID-19, other viral or mixed).

Pneumonia and clinical severity. CURB-65 scores,[2] Pneumonia severity index (PSI) scores,[3] multilobar pneumonia, pleural effusion, mechanical ventilation, ICU admission, steroid treatment, sepsis, severe sepsis, septic shock, acute respiratory distress syndrome (ARDS), and dialysis.

Delirium/Encephalopathy assessment methods: The Diagnostic and Statistics Manual-based assessment (DSM-IV or DSM-5).[4] Validated delirium scales included the confusion assessment method framework (CAM, CAM-ICU or other CAM-based method),[5] the Intensive Care Delirium Screening Checklist (ICDSC),[6] the Delirium Rating Scale-revised (DRS-R-98),[7] the 4 A's delirium assessment tool (4AT), the Nursing Delirium Screening Scale (NuDESC),[8] the Delirium Observation Scale (DOS),[9] and CHART-DEL[10]. Other non-delirium standardized assessments of mental status (AMS) included: the Richmond Agitation-Sedation Scale (RASS),[11] the Glasgow Coma Scale,[12] the Abbreviated Mental Test,[13] and the West-Haven criteria for hepatic encephalopathy.[14] We separately identified mental status ascertainment based on International Classification of Diseases (ICD) codes or if delirium was identified through non-structured, symptom-based reports (e.g. symptoms collection such as confusion, altered mental status, or altered consciousness).

Delirium characteristics: periods of delirium assessment, delirium subtypes (hyperactive, hypoactive, or mixed), delirium onset, or other factors associated with delirium.

Clinical outcomes: Length of hospitalization and stay in the ICU, length of ventilation, and mortality. For mortality we recorded the odds ratio results of any univariate or multivariable analyses.

Supplementary Tables

Table S1. Search Strategies across databases.

		**Ovid Medline**
**#**	**Search**	**Result**
1	exp Pneumonia/	374039
2	(Pneumonia* or Lung-inflammation or Inflammatory-lung-disease or pneumoniae* or Pneumonitis or Pneumonic or Respiratory-tract-infection* or Respiratory-infection* or Bronchopneumonia* or Bronchopneumoniae*).ti,ab.	289087
3	1 or 2	577636
4	exp Delirium/	13577
5	(delirium* or deliria or delirious-state* or clouding-of-consciousness or Encephalopathy).ti,ab.	78854
6	((acute or organic) adj2 (confusion or brain-syndrome or brain-dysfunction or brain-failure)).ti,ab.	1473
7	((altered) adj2 (mental-state or mental-status)).ti,ab.	5108
8	4 or 5 or 6 or 7	86201
9	exp Hospitalization/	304394
10	(inpatient* or in-patient* or hospitalise* or hospitalisation* or hospitalize* or hospitalization*).ti,ab.	2603655
11	9 or 10	2754518
12	3 and 8 and 11	1349
		**Cochrane CENTRAL**
#1	MeSH descriptor: [Pneumonia] explode all trees	12925
#2	(Pneumonia* or Lung-inflammation or Inflammatory-lung-disease or pneumoniae* or Pneumonitis or Pneumonic or Respiratory-tract-infection* or Respiratory-infection* or Bronchopneumonia* or Bronchopneumoniae*):ti,ab,kw	36311
#3	#1 OR #2	43138
#4	MeSH descriptor: [Delirium] explode all trees	1613
#5	(delirium* or deliria or delirious-state* or clouding-of-consciousness or Encephalopathy):ti,ab,kw	10501
#6	((acute or organic) NEAR/2 (confusion or brain-syndrome or brain-dysfunction or brain-failure)):ti,ab,kw	207
#7	((altered) NEAR/2 (mental-state or mental-status)):ti,ab,kw	238
#8	#4 OR #5 OR #6 OR #7	10860
#9	MeSH descriptor: [Hospitalization] explode all trees	20543
#10	(inpatient* or in-patient* or hospitalise* or hospitalisation* or hospitalize* or hospitalization*):ti,ab,kw	514079
#11	#9 OR #10	520134
#12	#3 AND #8 AND #11	400
		**Embase (Elsevier)**
1	‘pneumonia’/exp	
2	(Pneumonia* or Lung-inflammation or Inflammatory-lung-disease or pneumoniae* or Pneumonitis or Pneumonic or Respiratory-tract-infection* or Respiratory-infection* or Bronchopneumonia* or Bronchopneumoniae*):ti,ab	440183
3	#1 OR #2	641745
4	‘delirium’/exp	47236
5	(delirium* or deliria or delirious-state* or clouding-of-consciousness or Encephalopathy):ti,ab	125904
6	((acute or organic) NEAR/2 (confusion or brain-syndrome or brain-dysfunction or brain-failure)):ti,ab	2359
7	((altered) NEAR/2 (mental-state or mental-status)):ti,ab	11002

8	#4 OR #5 OR #6 OR #7	152921
9	'hospital patient'/exp	254138
10	(inpatient* or in-patient* or hospitalise* or hospitalisation* or hospitalize* or hospitalization):ti,ab	4132780
11	#9 OR #10	4159905
12	3 and 8 and 11	3136
		**PsycINFO (EBSCO)**
S1	DE "Pneumonia"	1015
S2	TI (Pneumonia* or Lung-inflammation or Inflammatory-lung-disease or pneumoniae* or Pneumonitis or Pneumonic or Respiratory-tract-infection* or Respiratory-infection* or Bronchopneumonia* or Bronchopneumonia*) OR AB (Pneumonia* or Lung-inflammation or Inflammatory-lung-disease or pneumoniae* or Pneumonitis or Pneumonic or Respiratory-tract-infection* or Respiratory-infection* or Bronchopneumonia* or Bronchopneumoniae*)	4153
S3	S1 OR S2	4921
S4	DE "Delirium"	14199
S5	TI (delirium* or deliria or delirious-state* or clouding-of-consciousness or Encephalopathy) OR AB (delirium* or deliria or delirious-state* or clouding-of-consciousness or Encephalopathy)	1160
S6	TI ((acute or organic) N2 (confusion or brain-syndrome or brain-dysfunction or brain-failure)) OR AB ((acute or organic) N2 (confusion or brain-syndrome or brain-dysfunction or brain-failure))	474
S7	TI ((altered) N2 (mental-state or mental-status)) OR AB ((altered) N2 (mental-state or mental-status))	15937
S8	S4 OR S5 OR S6 OR S7	15164
S9	DE "Hospitalized Patients"	852258
S10	TI (inpatient* or in-patient* or hospitalise* or hospitalisation* or hospitalize* or hospitalization) OR AB (inpatient* or in-patient* or hospitalise* or hospitalisation* or hospitalize* or hospitalization)	
S11	S9 OR S10	853076
S12	S3 AND S8 AND S11	165
		**Scopus (Elsevier)**
1	(TITLE-ABS (pneumonia* OR lung-inflammation OR inflammatory-lung-disease OR pneumoniae* OR pneumonitis OR pneumonic OR respiratory-tract-infection* OR respiratory-infection* OR bronchopneumonia* OR bronchopneumoniae*)) AND ((TITLE-ABS (delirium* OR deliria OR delirious-state* OR clouding-of-consciousness OR encephalopathy)) OR (TITLE-ABS ((altered) W/2 (mental-state OR mental-status))) OR (TITLE-ABS ((acute OR organic) W/2 (confusion OR brain-syndrome OR brain-dysfunction OR brain-failure)))) AND (TITLE-ABS (inpatient* OR in-patient* OR hospitalise* OR hospitalisation* OR hospitalize* OR hospitalization*))	868

Table S2. Number of papers retrieved from the databases searched.

Databases searched	Date searched	Results
Ovid MEDLINE	Aug/06/2024	1349
Cochrane Central Register of Controlled Trials	Aug06/2024	400
Embase	Aug/06/2024	3136
PsycINFO	Aug/06/2024	165
Scopus	Aug/06/2024	868
Total		5918
After de-duplication		4189

Table S3. Sensitivity Analysis Grading Criteria. JBI Critical Appraisal Checklist for Analytical Cross-Sectional Studies.

**1.**	**Were the criteria for inclusion in the sample clearly defined?**<br>*Description: The authors should provide clear inclusion and exclusion criteria that they developed prior to recruitment of the study participants. The inclusion/exclusion criteria should be specified (e.g., risk, stage of disease progression) with sufficient detail and all the necessary information critical to the study.*
**Yes**:	As close to all pneumonia patients as possible. OK if limited to certain patients such as specific pneumonia subtype (COVID-19, influenza, etc.), OK if limited to specific setting (ICU, Respiratory ward, postoperative), and OK if limited to certain age, but if there were too many other exclusions downgrade to "No"
**2.**	**Were the study subjects and the setting described in detail?**<br>*Description: The study sample should be described in sufficient detail so that other researchers can determine if it is comparable to the population of interest to them. The authors should provide a clear description of the population from which the study participants were selected or recruited, including demographics, location, and time period.*
**Yes**:	Clearly indicated demographics for study cohort, as well as where and when study was conducted.
**3.**	**Was the exposure (Pneumonia) measured in a valid and reliable way?**<br>*Description: The study should clearly describe the method of measurement of exposure. Assessing validity requires that a 'gold standard' is available to which the measure can be compared. The validity of exposure measurement usually relates to whether a current measure is appropriate or whether a measure of past exposure is needed. Reliability refers to the processes included in an epidemiological study to check repeatability of measurements of the exposures. These usually include intra-observer reliability and inter-observer reliability.*
**Yes:**	Clear process for diagnosing pneumonia, combination of respiratory signs/symptoms, signs/symptoms of infection, and corroboration by imaging (CXR or CT).
**4.**	**Were objective, standard criteria used for measurement of the condition (pneumonia)?**<br>*Description: It is useful to determine if patients were included in the study based on either a specified diagnosis or definition. This is more likely to decrease the risk of bias. Characteristics are another useful approach to matching groups, and studies that did not use specified diagnostic methods or definitions should provide evidence on matching by key characteristics.*
**Yes:**	Clear description of chest imaging (CXR or CT).
**5.**	**Were confounding factors identified?**<br>*Description: Confounding has occurred where the estimated intervention exposure effect is biased by the presence of some difference between the comparison groups (apart from the exposure investigated/of interest). Typical confounders include baseline characteristics, prognostic factors, or concomitant exposures (e.g., smoking). A confounder is a difference between the comparison groups, and it influences the direction of the study results. A high-quality study at the level of cohort design will identify the potential confounders and measure them (where possible). This is difficult for studies where behavioral, attitudinal or lifestyle factors may impact on the results.*
**Yes**:	Report of at least two of the following: Age, Dementia, Intubation/Mechanical Ventilation, ICU.
**6.**	**Were strategies to deal with confounding factors stated?**<br>*Description: Strategies to deal with effects of confounding factors may be dealt within the study design or in data analysis. By matching or stratifying sampling of participants, effects of confounding factors can be adjusted for. When dealing with adjustment in data analysis, assess the statistics used in the study. Most will be some form of multivariate regression analysis to account for the confounding factors measured.*
**Yes**:	Mulitvariable model or stratified approach with confounders included as independent variables and delirium as the dependent variable.
**7.**	**Were the outcomes (Delirium) measured in a valid and reliable way?**<br>*Description: If for e.g., lung cancer is assessed based on existing definitions or diagnostic criteria, then the answer to this question is likely to be yes. If lung cancer is assessed using observer reported, or self-reported scales, the risk of over- or under-reporting is increased, and objectivity is compromised. Importantly, determine if the measurement tools used were validated instruments as this has a significant impact on outcome assessment validity. Having established the objectivity of the outcome measurement (e.g., lung cancer) instrument, it's*

	important to establish how the measurement was conducted. Were those involved in collecting data trained or educated in the use of the instrument/s? (e.g., radiologists). If there was more than one data collector, were they similar in terms of level of education, clinical or research experience, or level of responsibility in the piece of research being appraised?
**Yes:**	Either prospective DSM based diagnosis by expert or validated clinical scale (CAM, CAM-ICU, ICSDC, 4AT, DRS, DOS, West Haven Criteria, CHART-DEL).
**8.**	**Was appropriate statistical analysis used?**<br>*Description: As with any consideration of statistical analysis, consideration should be given to whether there was a more appropriate alternate statistical method that could have been used. The methods section should be detailed enough for reviewers to identify which analytical techniques were used (in particular, regression or stratification) and how specific confounders were measured. For studies utilizing regression analysis, it is useful to identify if the study identified which variables were included and how they related to the outcome. If stratification was the analytical approach used, were the strata of analysis defined by the specified variables? Additionally, it is also important to assess the appropriateness of the analytical strategy in terms of the assumptions associated with the approach as differing methods of analysis are based on differing assumptions about the data and how it will respond.*
**Yes**:	If included report of how statistical analysis was used, given “Yes” if good description with multivariable regression modeling or at least basic counts.

Adapted from: Moola S, Munn Z, Tufanaru C, Aromataris E, Sears K, Sfetcu R, Currie M, Qureshi R, Mattis P, Lisy K, Mu P-F. Chapter 7: Systematic reviews of etiology and risk.[15]

Table S4. Included studies and their characteristics *(part 1)*

Study ID	Location	Sample size (n)	Hospital Setting	PNA Origin	Microbiology	Delirium Assessment	Age Mean ± SD or Median (IQR 25-75)	Delirium cases
Aliberti 2015[16]	Europe	172	Hospital wards	NS	Bacterial & Viral non-COVID	Delirium Scale	75 (66– 81)	53
Aliyu 2003[17]	North America	67	ED	CAP	NS	Symp. Collection	37 ± 8.76<br>Min, Max (18, 49)	0
Andrea 2024[18]	South America	191	mixed (ICU/non-ICU)	NS	Viral COVID	Symp. Collection	70.42 ± 7.7	48
Aziz 2018[19]	North America	18036	Hospital wards	NS	NS	ICD		488
Beretta 2023[20]	Europe	1357	Hospital wards	NS	Viral COVID	Symp. Collection		354
Bhansali 2021[21]	Asia	43	Hospital wards	NS	Viral COVID	Symp. Collection	Min 60	12
Bianchetti 2020[22]	Europe	82	Hospital wards	CAP	Viral COVID	Symp. Collection	82.6 ± 5.3	55
Blagoeva 2024[23]	Europe	144	Hospital wards	NS	Viral COVID	Symp. Collection	67 ± 14.7<br>Min 25, Max 92	11
Blot 2014[24]	Europe	192	Hospital wards	Mixed	Bacterial & Viral non-COVID	Symp. Collection	68.9 ± 18.8	39
Callan 2024[25]	North America	183	Hospital wards	HCAP	NS	Symp. Collection		86
Callea 2022[26]	Europe	71	Hospital wards	NS	Viral COVID	DSM	77 (68–82)	20
Capuzzi 2023[27]	Europe	221	Hospital wards	NS	Viral COVID	Symp. Collection	67.4 ± 14.5	54
Carr 2019[28]	North America	102	ICU	HCAP	NS	Symp. Collection	48 ± 17	73
Cataneo-Pina 2023[29]	North America	48	Hospital wards	CAP	Viral COVID; NA (CAP not specified)	Delirium Scale		25
Ceriani 2022[30]	Europe	110	Hospital wards	NS	Viral COVID	Delirium Scale	81 (79–84)	65
Chang 2024[31]	North America	1138	Hospital wards	NS	Viral COVID	Symp. Collection	65.4 ± 16.5	207
Chen 2020[32]	Asia	274	Hospital wards	NS	Viral COVID	Symp. Collection	62 (44–70)	26
Clemente 2002[33]	Europe	226	ED	CAP	NS	Symp. Collection	78.71<br>Min, Max (65, 96)	62
Cooper 2020[34]	North America	27	ICU	NS	Viral COVID	Delirium Scale	70 (54–76)	11
D'Ardes 2021[35]	Europe	56	Hospital wards	CAP	Viral COVID	Delirium Scale		14
Damanti 2023[36]	Europe	50	Hospital wards	NS	Viral COVID	Delirium Scale	79 (73-85)	13

Study ID	Location	Sample size (n)	Hospital Setting	PNA Origin	Microbiology	Delirium Assessment	Age Mean ± SD or Median (IQR 25-75)	Delirium cases
deHaan 2023[37]	Europe	175	Hospital wards	NS	NS	DSM		80
Denke 2018[38]	Europe	42	ICU	CAP	Bacterial & Viral non-COVID	Delirium Scale	Md 42 Min, Max (18, 65)	37
Diaz Fuenzalida 1999[39]	South America	96	Hospital wards	CAP	Bacterial	Symp. Collection	82.3 ± 8.3	48
Dravid 2021[40]	Asia	515	mixed (ICU/non-ICU)	CAP	Viral COVID	Symp. Collection	57 (46.5–66)	44
Eggers 2004[41]	Europe	20	ICU	HCAP	NS	DSM		11
Ekmekyapar 2022[42]	Asia	550	ICU	NS	Viral COVID	Delirium Scale		64
Fernandez-Sabe 2003[43]	Europe	1474	Hospital wards	CAP	Bacterial & Viral non-COVID	Symp. Collection	65.17 Min, max (16, 97)	189
Fimognari 2022[44]	Europe	318	Hospital wards	Mixed	NS	Symp. Collection		69
Garcia 2021[45]	North America	1040	Hospital wards	NS	Viral COVID	Symp. Collection	55.48 ± 14.47	224
Garcia-Grimshaw 2022[46]	North America	1017	mixed (ICU/non-ICU)	NS	Viral COVID	Delirium Scale		166
Garcia-Vidal 2008[47]	Europe	2457	Hospital wards	CAP	Bacterial	Symp. Collection	65.38 ± 16.85	314
Ghaffari 2021[48]	Asia	233	Hospital wards	NS	Viral COVID	Symp. Collection	65.21 ± 16.51	31
Gholi 2022[49]	Asia	310	ICU	NS	Viral COVID	Delirium Scale	73 ± 7	217
Gil 2006[50]	South America	1194	Hospital wards	CAP	NS	Symp. Collection	68 ± 17	270
Gogol 2011[51]	Europe	81937	Hospital wards	CAP	NS	Symp. Collection		6906
GomezDuque 2023[52]	South America	283	ICU	NS	Viral COVID	Delirium Scale	61.31 ± 13.8	148
Goncalves 2023[53]	South America	1874	mixed (ICU/non-ICU)	NS	Viral COVID	Symp. Collection	66.7 ± 10.4	744
Goss 2003[54]	North America	425	Hospital wards	CAP	NS	Symp. Collection	46 (18–100)	29
Guimaraes 2023[55]	South America	27	Hospital wards	Mixed	NS	AMS		14
Gupta 2024[56]	North America	15211	Hospital wards	CAP	NS	Symp. Collection	69.5 (58-81)	1080
Gutowski 2023[57]	Europe	335	mixed (ICU/non-ICU)	NS	Viral COVID	Symp. Collection	65.9 ± 15.2	72

Study ID	Location	Sample size (n)	Hospital Setting	PNA Origin	Microbiology	Delirium Assessment	Age Mean ± SD or Median (IQR 25-75)	Delirium cases
Hai 2024[58]	Asia	375	Hospital wards	NS	Viral COVID	Symp. Collection	59.4 ± 16.3	11
Helms 1979[59]	North America	57	Hospital wards	CAP	Bacterial	Symp. Collection	43.05 ± 3.58 Min, Max (19, 76)	9
Helms 2020[60]	Europe	140	ICU	NS	Viral COVID	Delirium Scale	62 (52–70)	118
Hoogewerf 2006[61]	Europe	260	Hospital wards	CAP	Bacterial	Symp. Collection		75
Hwang 2020[62]	Asia	91	Hospital wards	Mixed	Bacterial	Symp. Collection	67.42 ± 14.58	39
Iribarren-Diarasarri 2023[63]	Europe	143	ICU	NS	Viral COVID	Delirium Scale	61.1 ± 16.2	63
Johnson 2000[64]	North America	148	Hospital wards	NS	Bacterial	Symp. Collection	M 72.9	42
Jolley 2023[65]	North America	5979	mixed (ICU/ non-ICU)	NS	Viral COVID	ICD	61.1 ± 17.02	273
Jones 1993[66]	North America	176	Hospital wards	NS	Bacterial	Symp. Collection	58 ± 17.34	66
Kaneko 2022[67]	Asia	112	ICU	NS	Viral COVID	Symp. Collection	59.3 ± 13.4	24
Kelly 2009[68]	Europe	80	ED	CAP	NS	Symp. Collection	74 (18–95)	17
Kolditz 2015[69]	Europe	3427	Hospital wards	CAP	NS	Symp. Collection	67.08 Min, Max (18, 102)	204
Laurichesse 2001[70]	Europe	37	Hospital wards	CAP	NS	Symp. Collection	Min, Max (3, 93)	3
Lee 2023[71]	Asia	111	ICU	NS	Viral COVID	DSM	64.1 ± 13.1	26
Leijte 2020[72]	Europe	769	Hospital wards	NS	Viral COVID	Symp. Collection	69.8 ± 13.8	41
Lima 2021[73]	South America	60	ED	NS	NS	Symp. Collection		26
Limpawattana 2016[74]	Asia	36	ICU	NS	NS	Delirium Scale		21
Lin 2008[75]	Asia	49	ICU	NS	NS	Delirium Scale		14
Lin 2010[76]	North America	7554302	Hospital wards	NS	NS	ICD		70372
Lin 2010[77]	North America	564821	Hospital wards	NS	NS	ICD		4511
Liu 2007[78]	Asia	302	Hospital wards	CAP	Bacterial	Symp. Collection	67.74 ± 10.6	35
Liu 2021[79]	Asia	238	Hospital wards	NS	Viral COVID	Symp. Collection	72 (67–80)	61
Liu 2024[80]	North America	4483	Hospital wards	NS	NS	ICD		161

Study ID	Location	Sample size (n)	Hospital Setting	PNA Origin	Microbiology	Delirium Assessment	Age Mean ± SD or Median (IQR 25-75)	Delirium cases
Loponen 2008[81]	Europe	22	Hospital wards	NS	NS	Symp. Collection		5
Luna 2016[82]	Multilocation	6205	Hospital wards	CAP	Bacterial & Viral non-COVID	Symp. Collection	66.5 ± 17.9	776
Manali 2011[83]	Europe	67	Hospital wards	CAP	NS	Symp. Collection	58.8 ± 18.1	7
Marrie 2002[84]	North America	1339	Hospital wards	CAP	NS	Symp. Collection	64.28 ± 17.33	231
Marrie 2005[85]	North America	2464	Hospital wards	CAP	Bacterial	Symp. Collection	67.69 ± 16.75	249
Marrie 2007[86]	North America	1935	Hospital wards	CAP	Bacterial	Symp. Collection	78 ± 11.8	385
Martinez 2021[87]	Multilocation	159	Hospital wards	HCAP	Bacterial	AMS	58.9 ± 12.2	69
Martins 2022[88]	South America	59	ICU	NS	Viral COVID	Symp. Collection	Md 65<br>Min, Max (24, 81)	29
Matkovska 2019[89]	Europe	30	Hospital wards	NS	NS	NS		30
Melchio 2021[90]	Europe	591	Hospital wards	CAP	NS	AMS		102
Mendes 2021[91]	Europe	235	Hospital wards	NS	Viral COVID	DSM	86.3 ± 6.5	48
Mendez 2021[92]	Europe	179	mixed (ICU/non-ICU)	NS	Viral COVID	Symp. Collection	57 (49–67)	8
Morandi 2021[93]	Europe	241	Hospital wards	NS	Viral COVID	Delirium Scale	77.5 (65.6–85)	39
Mortensen 2002[94]	North America	2287	Hospital wards	CAP	NS	Symp. Collection		238
Otani 2022[95]	Asia	149	ED	NS	Viral COVID	Symp. Collection		100
Ozlu 2013[96]	Asia	264	mixed (ICU/non-ICU)	NS	Bacterial & Viral non-COVID	Symp. Collection	47.74 ± 18.67	35
Penafiel 2023[97]	South America	710	mixed (ICU/non-ICU)	CAP	Viral COVID	Symp. Collection	59.5 (48-70)<br>Min 18, Max 100	120
Pieralli 2014[98]	Europe	443	Hospital wards	CAP	NS	Delirium Scale	81.8 ± 7.5	110
Portela-Sanchez 2021[99]	Europe	71	mixed (ICU/non-ICU)	NS	Viral COVID	Symp. Collection	Md 69<br>Min, Max (23, 91)	13
Prabhahar 2024[100]	Asia	68	ED	NS	NS	Symp. Collection		21
Premkumar 2019[101]	Asia	110	ICU	NS	Bacterial & Viral non-COVID	AMS	47.7 ± 13.31	67
Quah 2021[102]	Asia	315	Hospital wards	CAP	Bacterial & Viral COVID	Symp. Collection	71.4 ± 18	26
Regueiro-Mira 2015[103]	Europe	240	Hospital wards	Mixed	Bacterial	Symp. Collection	57.2 ± 15.4<br>54.5 (55–59)	17

Study ID	Location	Sample size (n)	Hospital Setting	PNA Origin	Microbiology	Delirium Assessment	Age Mean ± SD or Median (IQR 25-75)	Delirium cases
Riquelme 1997[104]	South America	101	Hospital wards	CAP	Bacterial	DSM	78 ± 8	45
Riquelme 2006[105]	South America	200	Hospital wards	CAP	Bacterial	Symp. Collection	63 ± 19	54
Rothberg 2013[106]	North America	69344	Hospital wards	CAP	NS	Symp. Collection		2975
Ruiz 2014[107]	Europe	399	Hospital wards	NS	Bacterial	Symp. Collection		39
Sabzwari 2014[108]	Asia	39	Hospital wards	NS	NS	Symp. Collection		20
Sakakibara 2022[109]	Asia	136	Hospital wards	NS	NS	Symp. Collection	M 75<br>Min, max (3, 98)	136
Saldias 2002[110]	South America	463	Hospital wards	CAP	Bacterial & Viral non-COVID	Symp. Collection	68.8 ± 18.7<br>Min, max (16, 101)	119
Serrano 2023[111]	Europe	1371	Hospital wards	CAP	Bacterial	Symp. Collection	M 56.7	123
Serrano Fernandez 2022[112]	Europe	2224	Hospital wards	CAP	Bacterial & Viral COVID	Symp. Collection		153
Shirakawa 2022[113]	Asia	669	Hospital wards	NS	NS	Symp. Collection	78 (71–82)	88
Soares 2022[114]	Europe	165	mixed (ICU/non-ICU)	NS	NS	Symp. Collection		50
Sorrell 2023[115]	Europe	264	Hospital wards	Mixed	Viral COVID	Symp. Collection		88
SousaMatias 2024[116]	South America	152	Hospital wards	CAP	Bacterial	Symp. Collection	Md 58	44
Suwanpasu 2016[117]	Asia	23	Hospital wards	CAP	NS	Delirium Scale	M 81.3	13
Szylinska 2020[118]	Europe	217	Hospital wards	NS	NS	DSM		86
Tasci 2022[119]	Asia	154	mixed (ICU/non-ICU)	NS	Viral COVID	AMS	72.3 ± 15	95
Thabet 2022[120]	Asia	1413	Hospital wards	Mixed	Bacterial	AMS		249
Ticinesi 2020[121]	Europe	852	Hospital wards	NS	Viral COVID	Delirium Scale	73 ± 14	94
Tomasi 2017[122]	South America	30	Hospital wards	CAP	NS	Delirium Scale		10
Trevisan 2023[123]	Europe	1160	Hospital wards	NS	Viral COVID	DSM		197
Tuma 2021[124]	South America	55	mixed (ICU/ non-ICU)	NS	Viral COVID	AMS	59.84 ± 13.8	43
Uginet 2021[125]	Europe	707	mixed (ICU/non-ICU)	NS	NS	Symp. Collection		31

Study ID	Location	Sample size (n)	Hospital Setting	PNA Origin	Microbiology	Delirium Assessment	Age Mean ± SD or Median (IQR 25-75)	Delirium cases
vanderKnaap 2024[126]	Europe	324	ICU	NS	Viral COVID	Symp. Collection	64 (57-72)	48
Viasus 2012[127]	Europe	348	Hospital wards	NS	Bacterial & Viral non-COVID	Symp. Collection	44 (33–55)	23
Vinogradov 2021[128]	Multilocation	30	mixed (ICU/non-ICU)	CAP	Viral COVID	DSM	48.93 ± 8.47	10
Viscogliosi 2016[129]	Europe	159	Hospital wards	CAP	NS	DSM	80 ± 9.1	43
Watts 2012[130]	North America	363	ED	CAP	Bacterial & Viral non-COVID	Symp. Collection	63.03 ± 17.96	40
Wrenn 2023[131]	North America	300	ED	CAP	NS	Symp. Collection	60 (43-72)	46
Xing 2020[132]	Asia	100	Hospital wards	NS	NS	DSM	66.5 ± 3.93	22
Yang 2020[133]	North America	2031	Hospital wards	NS	NS	ICD		127
Yang 2022[134]	North America	5761	Hospital wards	NS	NS	ICD		333
Yang 2023[135]	North America	834	Hospital wards	NS	NS	ICD		58
Yavuz 2021[136]	Asia	186	ED	CAP	NS	Symp. Collection	79.4 ± 8.7<br>Med 79, Range 65-104	36
Yenibertiz 2021[137]	Asia	45	Hospital wards	NS	NS	DSM		34
Yuksel 2021[138]	Asia	307	mixed (ICU/non-ICU)	NS	Viral COVID	Symp. Collection	68.02 ± 15.64	213
Zerbit 2022[139]	Europe	85	mixed (ICU/non-ICU)	NS	Viral COVID	Symp. Collection	60 (49-69)	6
Zhang 2018[140]	Asia	1902	ED	CAP	NS	Symp. Collection	73 (61–82)	65
Zukowska 2023[141]	Europe	33	Hospital wards	HCAP	NS	Symp. Collection		6

Abbreviations: Intensive Care Unit (ICU), Emergency Department (ED). Community Acquired Pneumonia (CAP), Healthcare Acquired Pneumonia (HCAP), Not Specified (NS). Diagnostic and Statistical Manual (DSM); Delirium Scales: Confusion Assessment Method (CAM, CAM-ICU), Delirium Observation Scale (DOS), 4 A's Delirium Assessment Tool (4AT), Intensive Care Delirium Screening Checklist (ICDSC), and chart-validated scale (CHART-DEL);AMS scales: Richmond Agitation-Sedation Scale (RASS), Glasgow Coma Scale (GCS), Abbreviated Mental Test, and West-Haven Criteria for Hepatic Encephalopathy. International Classification of Diseases (ICD code). Retrospective (Retros.) and Prospective (Pros.) ascertainment of symptoms.

Table S5. Included studies and their characteristics *(part 2)*

Study	Study type	Pneumonia Diagnosis Criteria	Delirium Assessment Method specifications	Delirium onset (*n*=patients)	
				On admission	During hosp.
Aliberti 2015	Retros.	Respiratory signs/symptoms (clinical); Infectious signs/symptoms (clinical); Chest XRay	DeliriumScale: CHART-DEL		
Aliyu 2003	Retros.	ICD code only	Symp. Collection		
Andrea 2024	Retros.	CT scan	Symp. Collection		
Aziz 2018	Retros.	ICD code only	ICD		
Beretta 2023	Mixed	Respiratory signs/symptoms (clinical); Infectious signs/symptoms (clinical); Chest XRay	Symp. Collection		
Bhansali 2021	Retros.	Respiratory signs/symptoms (clinical); Infectious signs/symptoms (clinical); Chest XRay	Symp. Collection		
Bianchetti 2020	Retros.	Respiratory signs/symptoms (clinical); Infectious signs/symptoms (clinical)	Symp. Collection	55	
Blagoeva 2024	Retros.	Respiratory signs/symptoms (clinical); Infectious signs/symptoms (clinical); Chest XRay; CT scan	Symp. Collection	11	
Blot 2014	Mixed	Respiratory signs/symptoms (clinical); Infectious signs/symptoms (clinical); Chest XRay	Symp. Collection		
Callan 2024	Retros.	Not described	Symp. Collection		86
Callea 2022	NA	Respiratory signs/symptoms (clinical); Infectious signs/symptoms (clinical); Chest XRay; CT scan	DSM + CAM-ICU and RASS	8	12
Capuzzi 2023	Retros.	Not described	Symp. Collection		
Carr 2019	Retros.	Respiratory signs/symptoms (clinical); Infectious signs/symptoms (clinical); Chest XRay	Symp. Collection		
Cataneo-Pina 2023	Retros.	Not described	DeliriumScale: 3D-CAM		
Ceriani 2022	Retros.	Not described	DeliriumScale: CHART-DEL		
Chang 2024	Retros.	Respiratory signs/symptoms (clinical); Chest XRay	Symp. Collection		
Chen 2020	Retros.	Not described	Symp. Collection		
Clemente 2002	Retros.	Respiratory signs/symptoms (clinical); Infectious signs/symptoms (clinical); Chest XRay	Symp. Collection		
Cooper 2020	Prosp.	Not described	DeliriumScale: ICDSC		
D'Ardes 2021	Prosp.	Respiratory signs/symptoms (clinical); Infectious signs/symptoms (clinical); CT scan	DeliriumScale: 4AT		14
Damanti 2023	Prosp.	COVID-BioB protocol	DeliriumScale: 4AT		
deHaan 2023	Mixed	Not described	DSM + DOS		

Study	Study type	Pneumonia Diagnosis Criteria	Delirium Assessment Method specifications	Delirium onset (*n*=patients)	
				On admission	During hosp.
Denke 2018	Prosp.	Not described	DeliriumScale: CAM-ICU; RASS		37
DiazFuenzalida 1999	Retros.	Respiratory signs/symptoms (clinical); Infectious signs/symptoms (clinical); Chest XRay	Symp. Collection		
Dravid 2021	Retros.	Respiratory signs/symptoms (clinical); Infectious signs/symptoms (clinical); Chest XRay; CT scan	Symp. Collection		
Eggers 2004	Mixed	Respiratory signs/symptoms (clinical); Infectious signs/symptoms (clinical); Chest XRay; Other: CDC Definitions for pneumonia	DSM		
Ekmekyapar 2022	Retros.	Not described	DeliriumScale: CAM-ICU		
Fernandez-Sabe 2003	Prosp.	Respiratory signs/symptoms (clinical); Infectious signs/symptoms (clinical); Chest XRay	Symp. Collection		
Fimognari 2022	Prosp.	Respiratory signs/symptoms (clinical); Infectious signs/symptoms (clinical); Chest XRay; CT scan	Symp. Collection	69	
Garcia 2021	Retros.	Chart based only	Symp. Collection		
Garcia-Grimshaw 2022	Retros.	CT scan	DeliriumScale: CAM-ICU		166
Garcia-Vidal 2008	Prosp.	Respiratory signs/symptoms (clinical); Infectious signs/symptoms (clinical); Chest XRay	Symp. Collection	314	
Ghaffari 2021	Retros.	Respiratory signs/symptoms (clinical); Infectious signs/symptoms (clinical); CT scan	Symp. Collection		
Gholi 2022	Prosp.	Respiratory signs/symptoms (clinical); Infectious signs/symptoms (clinical); CT scan	DeliriumScale: CAM-ICU		
Gil 2006	Prosp.	Respiratory signs/symptoms (clinical); Infectious signs/symptoms (clinical); Chest XRay	Symp. Collection		
Gogol 2011	Retros.	Not described	Symp. Collection		
GomezDuque 2023	Retros.	Not described	DeliriumScale: CAM-ICU		
Goncalves 2023	Mixed	Respiratory signs/symptoms (clinical); Chest XRay; CT scan	Symp. Collection		
Goss 2003	Prosp.	Respiratory signs/symptoms (clinical); Infectious signs/symptoms (clinical); Chest XRay	Symp. Collection	29	
GuimarÃ£es 2023	Prosp.	Respiratory signs/symptoms (clinical); Infectious signs/symptoms (clinical); Chest XRay	AMS scale: West Haven criteria for hepatic encephalopathy	7	7
Gupta 2024	Mixed	Respiratory signs/symptoms (clinical); Infectious signs/symptoms (clinical); Chest XRay; CT scan	Symp. Collection		
Gutowski 2023	Retros.	Severe COVID	Symp. Collection		

Study	Study type	Pneumonia Diagnosis Criteria	Delirium Assessment Method specifications	Delirium onset (*n*=patients)	
				On admission	During hosp.
Hai 2024	NA	Respiratory signs/symptoms (clinical); Infectious signs/symptoms (clinical); Chest XRay	Symp. Collection		
Helms 1979	Retros.	Respiratory signs/symptoms (clinical); Infectious signs/symptoms (clinical); Chest Xray	Symp. Collection		
Helms 2020	Prosp.	Respiratory signs/symptoms (clinical); Infectious signs/symptoms (clinical); CT scan	DeliriumScale: CAM-ICU; RASS	22	97
Hoogewerf 2006	Prosp.	Respiratory signs/symptoms (clinical); Infectious signs/symptoms (clinical); Chest XRay	Symp. Collection		
Hwang 2020	Retros.	Chart based only	Symp. Collection		
Iribarren-Diarasarri 2023	Prosp.	Not described	DeliriumScale: CAM-ICU		
Johnson 2000	Retros.	ICD code only	Symp. Collection		
Jolley 2023	Retros.	Chart based only	ICD		
Jones 1993	Retros.	Chest XRay; Chart based only	Symp. Collection		
Kaneko 2022	Retros.	Not described	Symp. Collection		
Kelly 2009	Prosp.	Respiratory signs/symptoms (clinical); Infectious signs/symptoms (clinical); Chest XRay	Symp. Collection		
Kolditz 2015	Prosp.	Respiratory signs/symptoms (clinical); Infectious signs/symptoms (clinical); Chest XRay; ATS/IDSA 2007 minor criteria for severe pneumonia	Symp. Collection		
Laurichesse 2001	Prosp.	Respiratory signs/symptoms (clinical); Infectious signs/symptoms (clinical); Chest XRay	Symp. Collection		
Lee 2023	Retros.	Not described	DSM + CAM-ICU and RASS		
Leijte 2020	Retros.	Respiratory signs/symptoms (clinical); Infectious signs/symptoms (clinical); CT scan	Symp. Collection	41	
Lima 2021	Prosp.	Not described	Symp. Collection		26
Limpawattana 2016	Prosp.	Not described	DeliriumScale: CAM-ICU		
Lin 2008	NA	Not described	DeliriumScale: CAM-ICU; RASS; GCS		
Lin 2010	Retros.	Diagnosis-related groups [DRGs] categories for pneumonia	ICD		
Lin 2010	Retros.	Center for Medicare and Medicaid Services DRG (CMS-DRG classifications) categories fro pneumonia	ICD	3241	668

Study	Study type	Pneumonia Diagnosis Criteria	Delirium Assessment Method specifications	Delirium onset (*n*=patients)	
				**On admission**	**During hosp.**
Liu 2007	Retros.	Respiratory signs/symptoms (clinical); Infectious signs/symptoms (clinical); Chest XRay	Symp. Collection		
Liu 2021	Retros.	Respiratory signs/symptoms (clinical); Infectious signs/symptoms (clinical)	Symp. Collection		
Liu 2024	Retros.	ICD code only	ICD		
Loponen 2008	Mixed	Not described	Symp. Collection		
Luna 2016	Retros.	Respiratory signs/symptoms (clinical); Infectious signs/symptoms (clinical); Chest Xray	Symp. Collection		
Manali 2011	Retros.	Respiratory signs/symptoms (clinical); Infectious signs/symptoms (clinical); Chest XRay; CT scan	Symp. Collection		
Marrie 2002	Prosp.	Respiratory signs/symptoms (clinical); Infectious signs/symptoms (clinical); Chest XRay	Symp. Collection		
Marrie 2005	Prosp.	Respiratory signs/symptoms (clinical); Infectious signs/symptoms (clinical); Chest XRay	Symp. Collection		
Marrie 2007	Prosp.	Respiratory signs/symptoms (clinical); Infectious signs/symptoms (clinical); Chest XRay	Symp. Collection		
Martinez 2021	Mixed	Infectious signs/symptoms (clinical); Chest Xray	AMS scale: hepatic encephalopathy grade III-IV		
Martins 2022	Retros.	Not described	Symp. Collection		
Matkovska 2019	NA	Not described	NA		
Melchio 2021	Mixed	Respiratory signs/symptoms (clinical); Infectious signs/symptoms (clinical); Chest Xray	AMS scale: Abbrev. mental test	102	
Mendes 2021	Retros.	Respiratory signs/symptoms (clinical); Infectious signs/symptoms (clinical); Chest XRay; CT scan	DSM + CAM	48	
Mendez 2021	Prosp.	Infectious signs/symptoms (clinical); Chest Xray	Symp. Collection		
Morandi 2021	Retros.	Infectious signs/symptoms (clinical); Chest XRay; CT scan; Recorded from the chart	DeliriumScale: 4AT and CHART-DEL	39	
Mortensen 2002	Prosp.	Respiratory signs/symptoms (clinical); Infectious signs/symptoms (clinical); Chest XRay	Symp. Collection		
Otani 2022	NA	Not described	Symp. Collection		
Ozlu 2013	Retros.	Respiratory signs/symptoms (clinical); Infectious signs/symptoms (clinical); Chest XRay	Symp. Collection		
Penafiel 2023	Prosp.	Respiratory signs/symptoms (clinical); Infectious signs/symptoms (clinical); Chest XRay	Symp. Collection		
Pieralli 2014	Retros.	Respiratory signs/symptoms (clinical); Infectious signs/symptoms (clinical); Chest XRay; CT scan	DeliriumScale: CAM		110

Study	Study type	Pneumonia Diagnosis Criteria	Delirium Assessment Method specifications	Delirium onset (*n*=patients)	
				On admission	During hosp.
Portela-Sanchez 2021	Prosp.	Infectious signs/symptoms (clinical); Chest XRay	Symp. Collection		
Prabhahar 2024	Retros.	Respiratory signs/symptoms (clinical); Infectious signs/symptoms (clinical); Chest XRay; CT scan	Symp. Collection		
Premkumar 2019	Prosp.	Respiratory signs/symptoms (clinical); Infectious signs/symptoms (clinical); Chest XRay	AMS		
Quah 2021	Mixed	Respiratory signs/symptoms (clinical); Infectious signs/symptoms (clinical); Chest XRay	Symp. Collection		
Regueiro-Mira 2015	Retros.	Respiratory signs/symptoms (clinical); Infectious signs/symptoms (clinical); Chest XRay; Criteria approved by the Spanish Society of Pulmonology and Thoracic Surgery (SEPAR). Confirmation by antigen determination of L. pneumophila in urine	Symp. Collection		
Riquelme 1997	Prosp.	Respiratory signs/symptoms (clinical); Infectious signs/symptoms (clinical); Chest XRay	DSM	45	
Riquelme 2006	Prosp.	Respiratory signs/symptoms (clinical); Infectious signs/symptoms (clinical); Chest XRay	Symp. Collection		
Rothberg 2013	Retros.	ICD code only	Symp. Collection		
Ruiz 2014	Prosp.	Respiratory signs/symptoms (clinical); Infectious signs/symptoms (clinical); Chest XRay	Symp. Collection		
Sabzwari 2014	Retros.	Not described	Symp. Collection		
Sakakibara 2022	Retros.	Not described	Symp. Collection		
Saldias 2002	Prosp.	Respiratory signs/symptoms (clinical); Infectious signs/symptoms (clinical); Chest XRay	Symp. Collection		
Serrano 2023	Prosp.	Respiratory signs/symptoms (clinical); Infectious signs/symptoms (clinical); Chest XRay; Other: urinary antigen tests	Symp. Collection		
SerranoFernandez 2022	Prosp.	Respiratory signs/symptoms (clinical); Infectious signs/symptoms (clinical); Chest XRay	Symp. Collection		
Shirakawa 2022	Retros.	ICD code only	Symp. Collection		
Soares 2022	Retros.	Stroke-associated pneumonia- based on CDC Criteria	Symp. Collection		
Sorrell 2023	Retros.	Not described	Symp. Collection		
SousaMatias 2024	Retros.	Respiratory signs/symptoms (clinical); Infectious signs/symptoms (clinical); Chest XRay	Symp. Collection		

Study	Study type	Pneumonia Diagnosis Criteria	Delirium Assessment Method specifications	Delirium onset (*n*=patients)	
				On admission	During hosp.
Suwanpasu 2016	Prosp.	Not described	DeliriumScale: CAM		
Szylinska 2020	Retros.	Not described	DSM + CAM-ICU		
Tasci 2022	Retros.	CT scan	AMS scale: RASS		
Thabet 2022	Prosp.	Respiratory signs/symptoms (clinical); Infectious signs/symptoms (clinical); Chest XRay; CT scan; ATS/IDSA citation	AMS scale: GCS		
Ticinesi 2020	Retros.	Respiratory signs/symptoms (clinical); Infectious signs/symptoms (clinical); CT scan	DeliriumScale: CAM shortened version		94
Tomasi 2017	Prosp.	Chart based only	DeliriumScale: CAM		
Trevisan 2023	Retros.	Chest XRay; CT scan	DSM + RASS		
Tuma 2021	Retros.	CT scan	AMS Scale:RASS		
Uginet 2021	Retros.	Respiratory signs/symptoms (clinical); Infectious signs/symptoms (clinical)	Symp. Collection		
vanderKnaap 2024	Prosp.	Respiratory signs/symptoms (clinical); Infectious signs/symptoms (clinical); CT scan	Symp. Collection		
Viasus 2012	Prosp.	Respiratory signs/symptoms (clinical); Infectious signs/symptoms (clinical); Chest XRay	Symp. Collection		
Vinogradov 2021	Prosp.	CT scan	DSM		
Viscogliosi 2016	Prosp.	Not described	DSM +CAM		
Watts 2012	Retros.	Chart based only	Symp. Collection		
Wrenn 2023	Retros.	Chart based only; ICD code only	Symp. Collection		
Xing 2020	NA	Not described	DSM		
Yang 2020	Retros.	ICD code only	ICD		
Yang 2022	Retros.	ICD code only	ICD		
Yang 2023	Retros.	ICD code only	ICD		
Yavuz 2021	Retros.	Respiratory signs/symptoms (clinical); Infectious signs/symptoms (clinical); Chest XRay	Symp. Collection		
Yenibertiz 2021	Retros.	Not described	DSM		
Yuksel 2021	Retros.	Respiratory signs/symptoms (clinical); Infectious signs/symptoms (clinical); Chest XRay; CT scan	Symp. Collection		
Zerbit 2022	Retros.	Respiratory signs/symptoms (clinical); Chest XRay; CT scan	Symp. Collection		
Zhang 2018	Retros.	Respiratory signs/symptoms (clinical); Infectious signs/symptoms (clinical); Chest Xray	Symp. Collection		

Study	Study type	Pneumonia Diagnosis Criteria	Delirium Assessment Method specifications	Delirium onset (*n*=patients)	
				On admission	During hosp.
Zukowska 2023	Retros.	Respiratory signs/symptoms (clinical); Infectious signs/symptoms (clinical); Chest XRay; CT scan; ECDC criteria	Symp. Collection		

Abbreviations: Not Specified (NA). Diagnostic and Statistical Manual (DSM); Delirium Scales: Confusion Assessment Method (CAM, CAM-ICU), Delirium Observation Scale (DOS), 4 A's Delirium Assessment Tool (4AT), Intensive Care Delirium Screening Checklist (ICDSC), and chart-validated scale (CHART-DEL);AMS scales: Richmond Agitation-Sedation Scale (RASS), Glasgow Coma Scale (GCS), Abbreviated Mental Test, and West-Haven Criteria for Hepatic Encephalopathy. International Classification of Diseases (ICD code), Symptom (Symp) Collection. Restrospective (Retros.), Prospective (Prosp.).

Table S6. Relationship of delirium to baseline characteristics, Comorbidities, measures of Pneumonia Severity and Acute Care, Mortality, and Length of Clinical Care in Studies at Low Risk of Bias.

**Baseline characteristics**	**OR**	**95% CI**	***p*-value**
Female (n=5)	0.77	[0.47; 1.25]	0.204
Nursing home (n=1)	2.33	[1.15; 4.74]	0.019*
Age	DOM +6.20	[1.49;10.92]	0.01*
**Comorbidities**	**OR**	**95% CI**	**p-value**
Dementia (n=3)	4.12	[1.91; 8.89]	0.015*
Stroke (n=4)	2.21	[1.31; 3.74]	0.017*
Respiratory disease (n=3)	1.97	[0.75; 5.14]	0.093
Liver Disease (n=1)	0.97	[0.04; 20.80]	0.982
Kidney Disease (n=3)	1.45	[0.08; 24.67]	0.631
Hypertension (n=3)	1.21	[0.33; 4.45]	0.601
Heart Disease (n=4)	0.78	[0.18; 3.51]	0.64
Diabetes (n=4)	1.11	[0.72; 1.72]	0.484
Cancer (n=3)	1.06	[0.08; 13.62]	0.933
**Pneumonia Severity and Acute Care**			
ICU Admission (n=3)	2.67	[0.57; 12.49]	0.112
Invasive Ventilation (n=1)	5.27	[0.10; 272.37]	0.409
Noninvasive Ventilation (n=2)	1.58	[0.00; 55214680.00]	0.794
Steroids (n=1)	2.07	[0.10; 45.04]	0.643
**Length of Clinical Care**	**DOM**	**95%CI**	***p*-value**
Length of Invasive ventilation (n = 1)	+6.00	[1.54; 10.46]	0.008*
Length of ICU stay (n = 1)	+4.67	[-0.65; 9.98]	0.085
Length of Hospitalization (n = 1)	+0.80	[-3.01; 1.41]	0.470
**Mortality**			
Overall Death (univariate analysis) (n=6)	3.70	[1.14; 12.05]	0.036*
Overall Death (multivariate analysis (n = 5)	2.16	[1.19; 3.91],	0.023*

All comorbidities reflect reports of chronic disease prior to admission. *n* = studies, OR = odds ratios, DOM = difference of means, CI = Confidence interval. Significant p-value <0.05 (*). Forest plots and measures of heterogeneity for each baseline characteristics, comorbidities, pneumonia severity factors and acute care, length of clinical care, and mortality for studies at low risk of bias are shown in Supplementary Figures S13, S14, S16, S18 and S19 respectively).

Supplementary Figures

Figure S1. Traffic light plot for risk of bias in individual studies

Risk of bias was assessed over eight domains (D1–D8). Overall risk of bias was classified as "low" *(green circles)* if studies met ≥6/8 domains including clear ascertainment of both pneumonia and delirium using standardized methods or validated clinical scores. Risk of bias was classified as "unclear" *(yellow circles)* if studies met 6/8 domains, but pneumonia and/or delirium diagnosis were not clearly ascertained, and as "high" *(red circles)* if they meet <6/8 domains and no clear ascertainment for pneumonia or delirium using standardized methods were used. Plot created using *robvis*, a web app built in R for visualizing risk-of-bias assessments.[142]

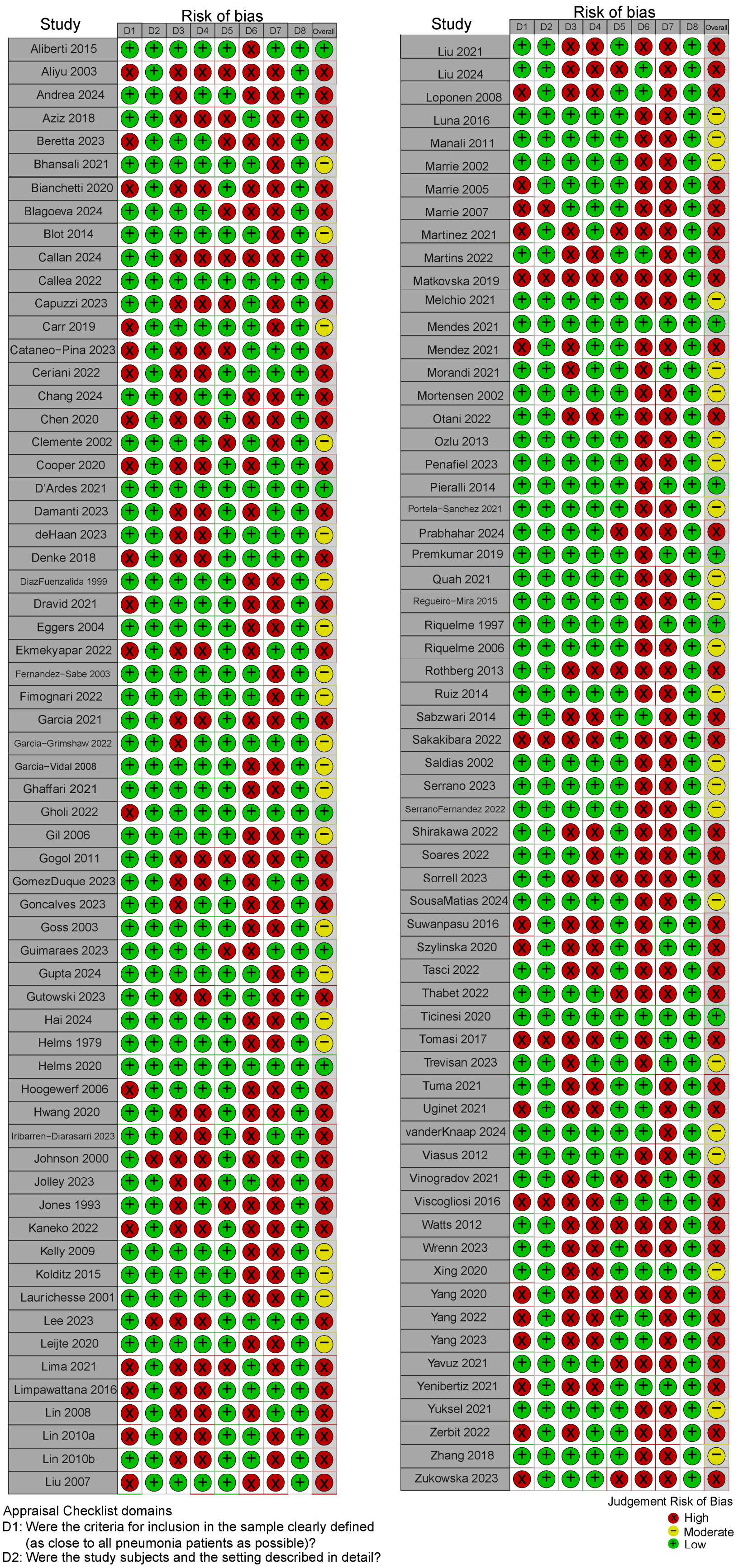

Appraisal Checklist domains
D1: Were the criteria for inclusion in the sample clearly defined
(as close to all pneumonia patients as possible)?
D2: Were the study subjects and the setting described in detail?
D3: Was the exposure (pneumonia) measured in a valid & reliable way?
D4: Were objective, standard criteria used for measurement of the condition (pneumonia)?
D5: Were confounding factors identified (at least 2/4 of Age, Dementia, ICU, mechanical ventilation?
D6: Were strategies to deal with confounding factors stated?
D7: Were the outcomes (delirium) measured in a valid & reliable way (prospective DSM-based diagnosis by expert
or validated clinical scale (CAM, CAM-ICU, ICDSC, 4AT, DRS, CHART-DEL, West Haven criteria)?
D8: Was the appropriate statistical analysis used?

Figure S2. Funnel plots for all included studies reflect true heterogeneity rather than publication (selection) bias.

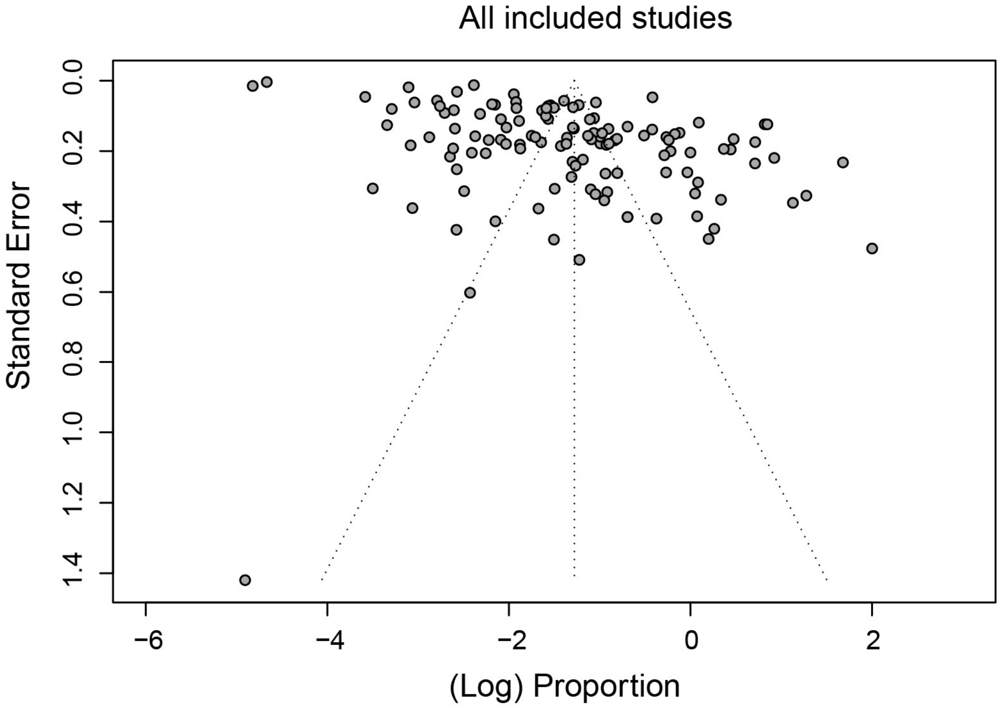

A. Assessment for potential publication bias, using ‘funnel plot’ of standard error by logistic event rate for all included studies.

Figure S3. Forest plot by risk of bias assessment.

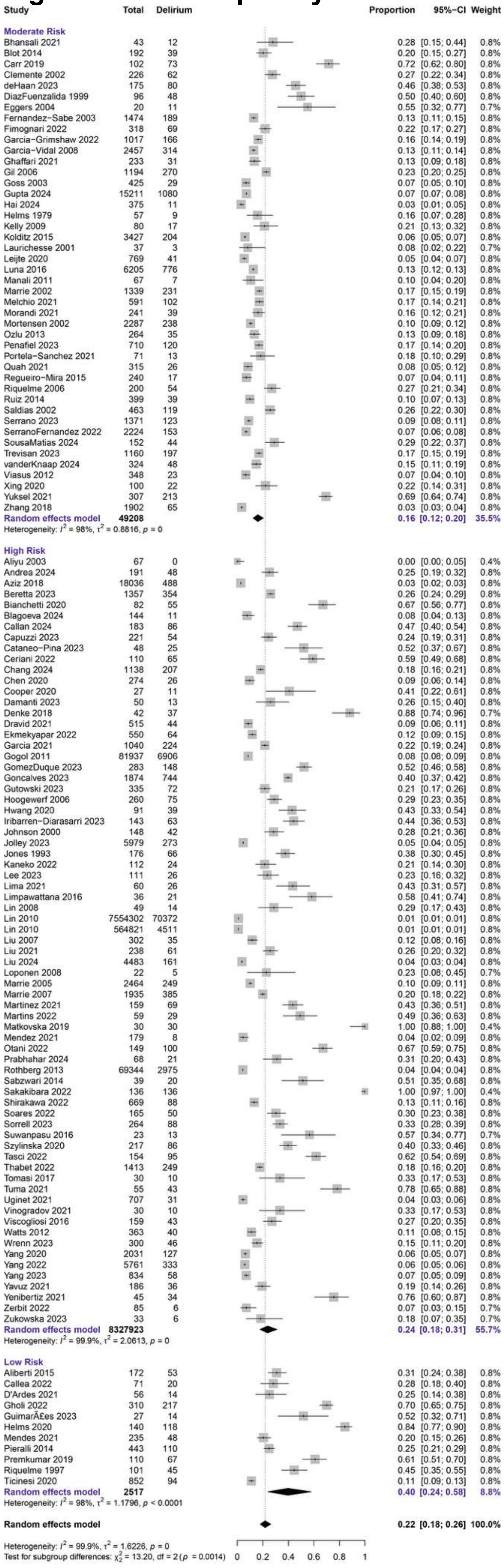

Delirium identification is increased in studies at low risk of bias using validated methods to diagnose delirium and pneumonia. Each paper was graded for risk of bias using the JBI manual for evidence synthesis tool across eight different quality measures (Supplementary Table S2 describes in detail the grading criteria used and Supplementary Figure S1 shows the grading for each primary study). Overall risk of bias was classified as "low" if studies met ≥6/8 domains including clear ascertainment of both pneumonia and delirium using standardized methods or validated clinical scores.

Figure S4. Forest plot by delirium assessment method

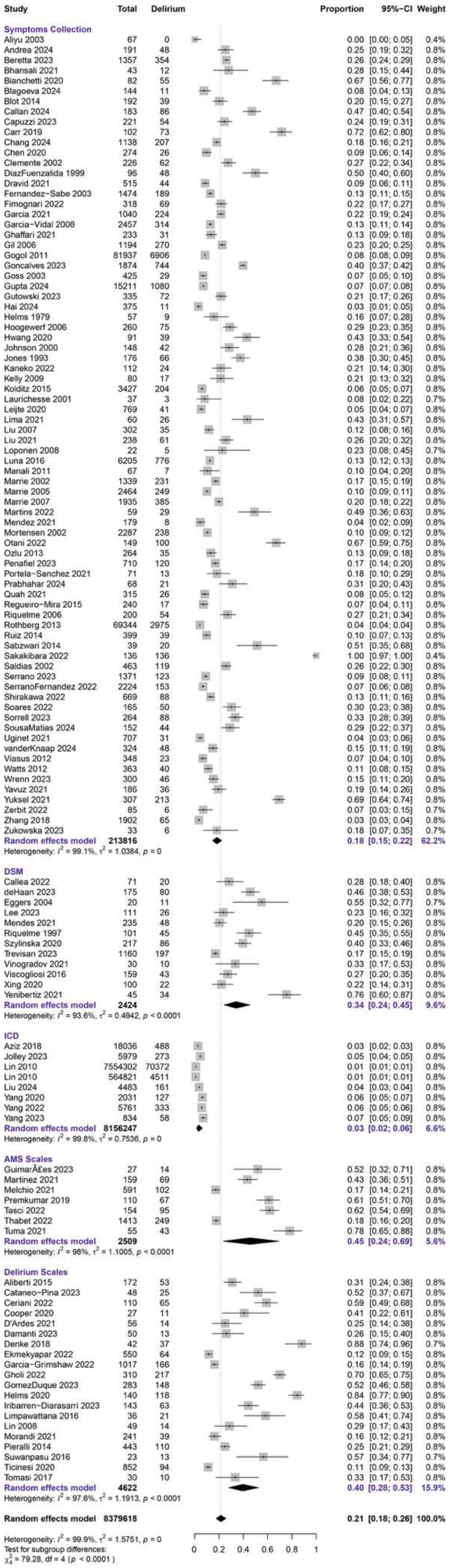

Figure S5. Forest plot by Hospital Setting

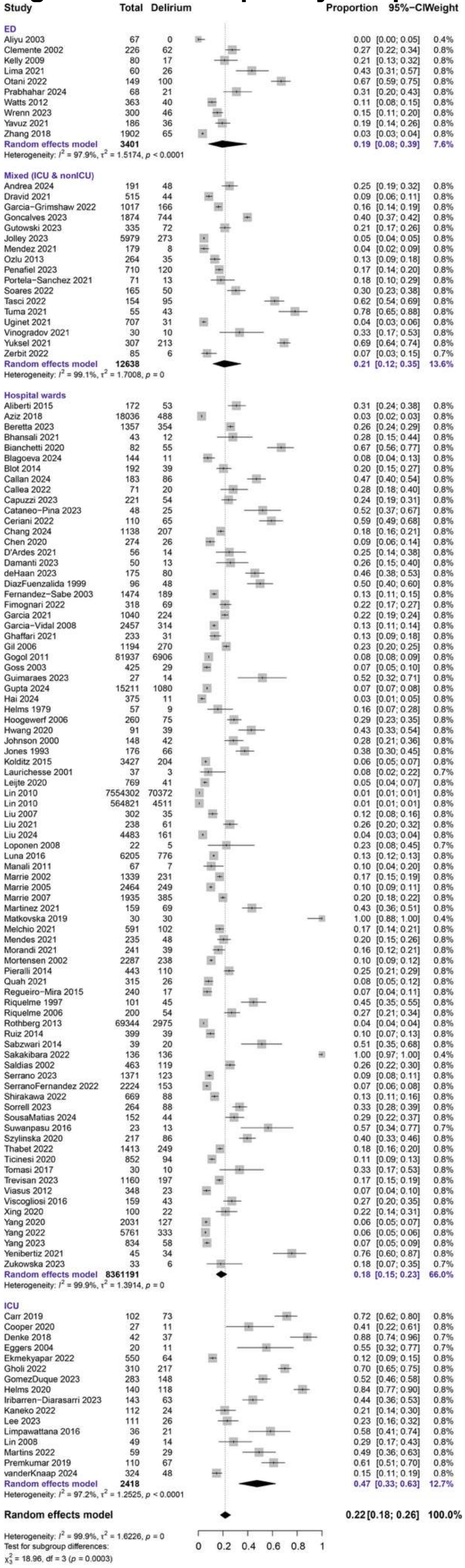

Figure S6. Forest plot by Pneumonia Infection Origin

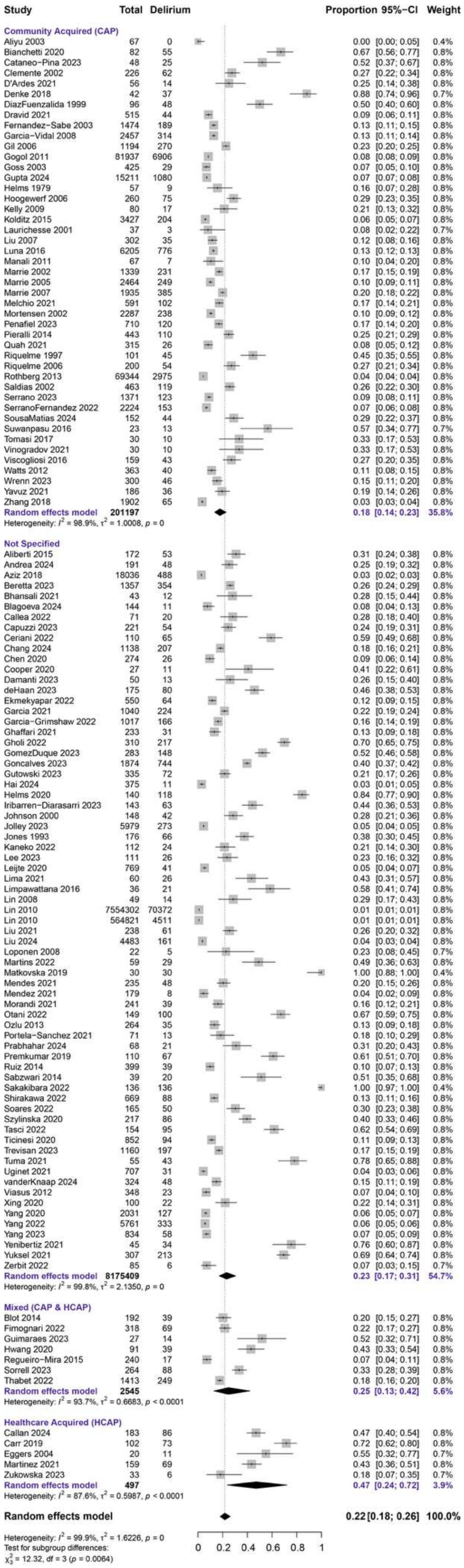

Figure S7. Forest plot by Microbiological etiology

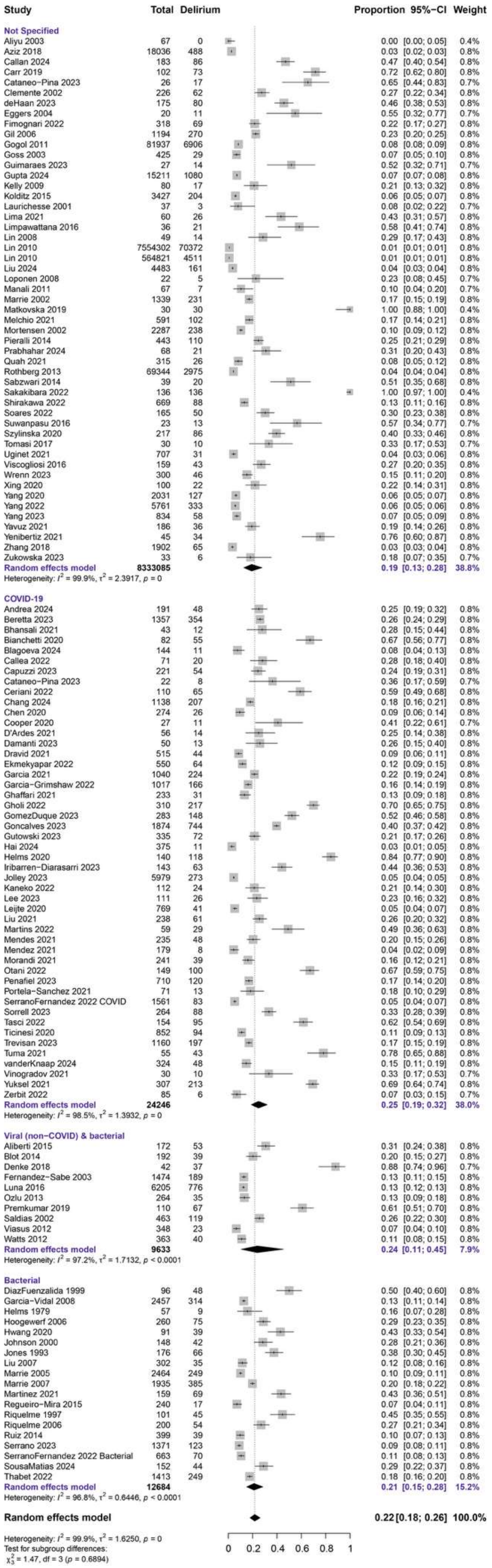

Figure S8. Subgroup analysis in studies at low risk of bias

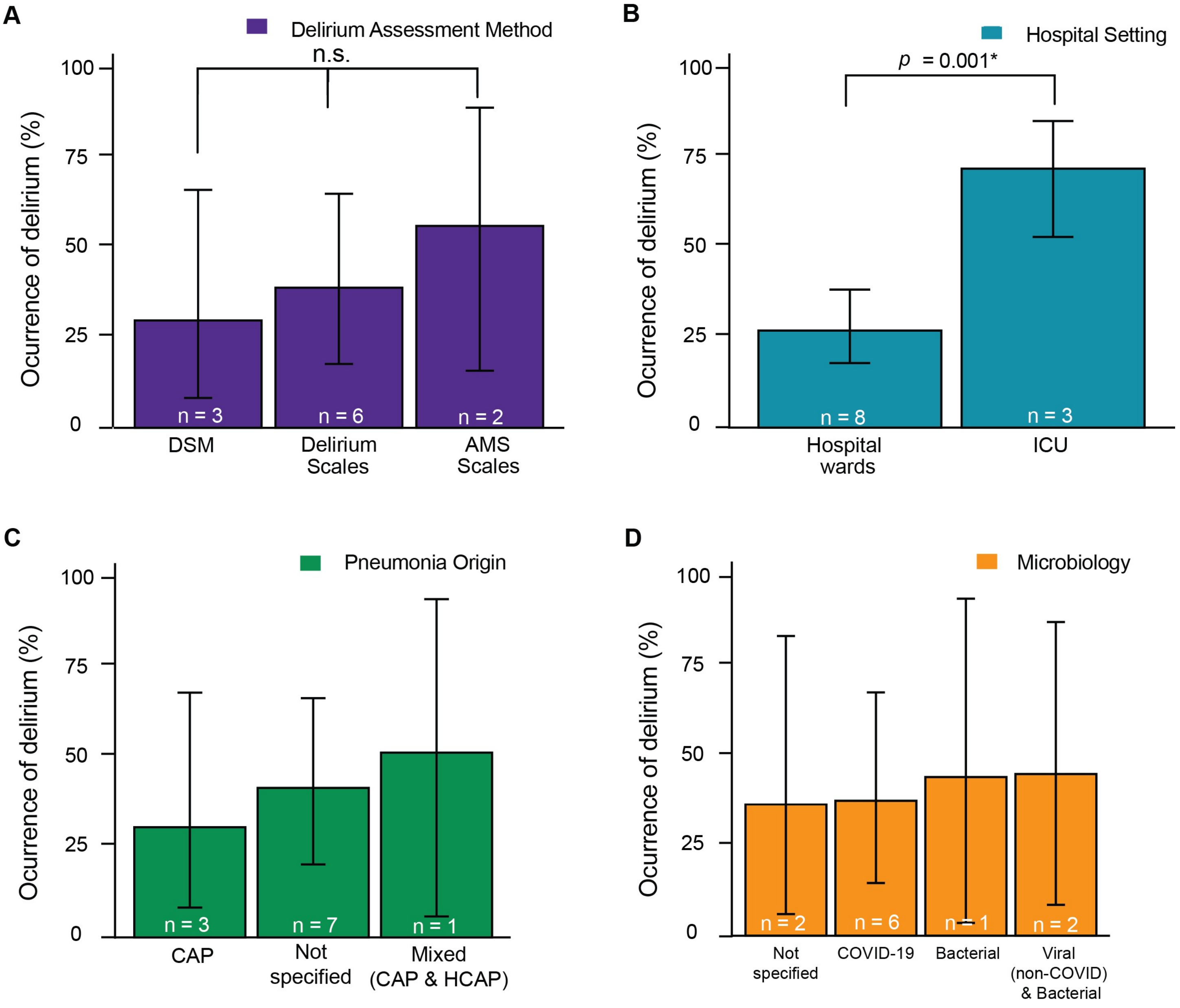

A. Ascertainment of delirium through validated methods was a criterion to classify studies as low risk of bias. As expected, delirium rates did not vary significantly according to the validated assessment method used in these studies (R^2 = 0%, p = 0.60). Compared to the DSM, delirium rates were similar when assessed using standardized assessments of mental status (AMS Scales, p=0.98) and with validated Delirium scales (p=0.59). Standardized assessments of mental status included the Glasgow Coma Scale, Richmond Agitation Sedation Scale, Abbreviated Mental Test, and West-Haven criteria for hepatic encephalopathy. Delirium scales included CAM, CAM-ICU, ICSDC, 4AT, DRS, DOS, and CHART-DEL (see abbreviations in Supplementary Methods). Each bar represents a meta-analytic estimate of delirium rates, with the calculated 95% confidence interval. Post-hoc p-values in all panels are adjusted for multiple comparisons (Holm). Forest plots of all studies with N, meta-analytic proportion, CI and heterogeneity measures per each subgroup analysis are provided in Supplementary Figure S9A.

B. In studies at low risk of bias, delirium rates varied significantly according to study setting, which explained 68.2% of variance in delirium rates across studies (R^2 68.2%, p=0.0015). Delirium rates were significantly higher for studies performed in the Intensive Care Unit (ICU) compared to those in hospital wards (p=0.001). Conventions as in A, with forest plots in Supplementary figure S9B).

C. Delirium rates did not vary significantly according to pneumonia origin in studies at low risk of bias, which 0% of variance in delirium rates across studies (R^2 = 0%, *p*=0.77). (CAP=Community Acquired Pneumonia, HCAP=Healthcare Acquired Pneumonia). Conventions as in A, with forest plots in Supplementary Figure S9C).
D. Delirium rates did not vary significantly according to microbiological etiologies in studies at low risk of bias, which explained 0.0% of variance in delirium rates across studies (R^2 = 0.0%, *p*=0.98). Conventions as in A, with forest plots in Supplementary Figure 9D).

Figure S9. Forest plots per subgroup analysis in studies at low risk of bias

A. Delirium Assessment method

Study	Total	Delirium	Proportion	95%−CI	Weight
**Delirium scale**					
Aliberti 2015	172	53	0.31	[0.24; 0.38]	9.2%
D'Ardes 2021	56	14	0.25	[0.14; 0.38]	8.8%
Gholi 2022	310	217	0.70	[0.65; 0.75]	9.3%
Helms 2020	140	118	0.84	[0.77; 0.90]	9.0%
Pieralli 2014	443	110	0.25	[0.21; 0.29]	9.4%
Ticinesi 2020	852	94	0.11	[0.09; 0.13]	9.4%
**Random effects model**	**1973**		**0.39**	**[0.13; 0.74]**	**55.1%**
Heterogeneity: $I^2 = 98.9\%$, $\tau^2 = 1.9413$, $p < 0.0001$					
**DSM**					
Callea 2022	71	20	0.28	[0.18; 0.40]	8.9%
Mendes 2021	235	48	0.20	[0.15; 0.26]	9.3%
Riquelme 1997	101	45	0.45	[0.35; 0.55]	9.1%
**Random effects model**	**407**		**0.30**	**[0.09; 0.65]**	**27.3%**
Heterogeneity: $I^2 = 89.8\%$, $\tau^2 = 0.3081$, $p < 0.0001$					
**AMS**					
Guimaraes 2023	27	14	0.52	[0.32; 0.71]	8.4%
Premkumar 2019	110	67	0.61	[0.51; 0.70]	9.2%
**Random effects model**	**137**		**0.59**	**[0.18; 0.91]**	**17.6%**
Heterogeneity: $I^2 = 0\%$, $\tau^2 = 0$, $p = 0.3924$					
**Random effects model**	**2517**		**0.40**	**[0.24; 0.58]**	**100.0%**

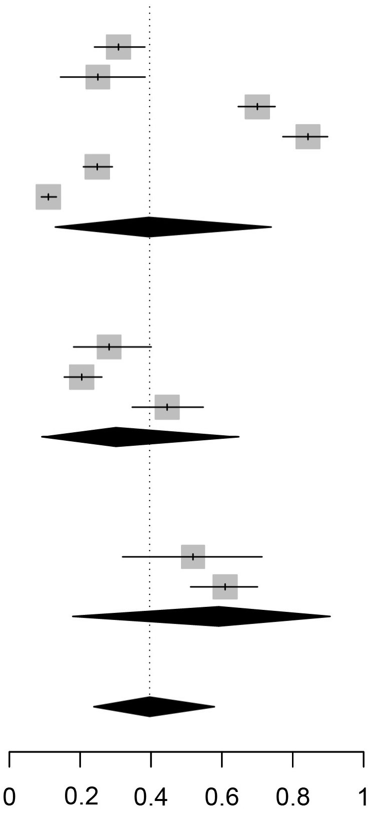

Heterogeneity: $I^2 = 98.0\%$, $\tau^2 = 1.1796$, $p < 0.0001$
Test for subgroup differences: $\chi^2_2 = 11.80$, df = 2 ($p = 0.0027$)

B. Hospital Setting

Study	Total	Delirium	Proportion	95%−CI	Weight
**Hospital wards**					
Aliberti 2015	172	53	0.31	[0.24; 0.38]	9.2%
Callea 2022	71	20	0.28	[0.18; 0.40]	8.9%
D'Ardes 2021	56	14	0.25	[0.14; 0.38]	8.8%
Guimaraes 2023	27	14	0.52	[0.32; 0.71]	8.4%
Mendes 2021	235	48	0.20	[0.15; 0.26]	9.3%
Pieralli 2014	443	110	0.25	[0.21; 0.29]	9.4%
Riquelme 1997	101	45	0.45	[0.35; 0.55]	9.1%
Ticinesi 2020	852	94	0.11	[0.09; 0.13]	9.4%
**Random effects model**	**1957**		**0.27**	**[0.18; 0.39]**	**72.5%**
Heterogeneity: $I^2 = 93.5\%$, $\tau^2 = 0.3853$, $p < 0.0001$					
**ICU**					
Gholi 2022	310	217	0.70	[0.65; 0.75]	9.3%
Helms 2020	140	118	0.84	[0.77; 0.90]	9.0%
Premkumar 2019	110	67	0.61	[0.51; 0.70]	9.2%
**Random effects model**	**560**		**0.73**	**[0.36; 0.93]**	**27.5%**
Heterogeneity: $I^2 = 88.2\%$, $\tau^2 = 0.3384$, $p = 0.0002$					
**Random effects model**	**2517**		**0.40**	**[0.24; 0.58]**	**100.0%**

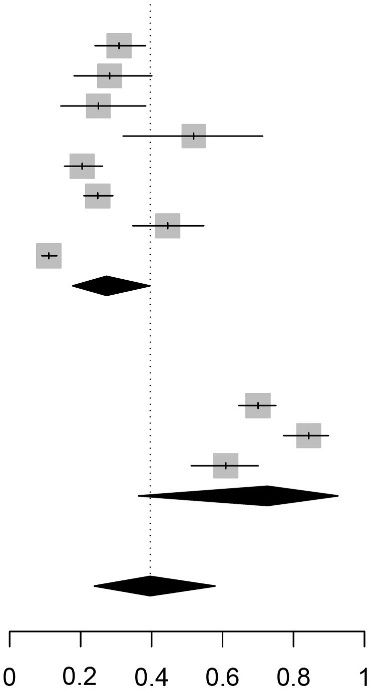

Heterogeneity: $I^2 = 98.0\%$, $\tau^2 = 1.1796$, $p < 0.0001$
Test for subgroup differences: $\chi^2_1 = 20.96$, df = 1 ($p < 0.0001$)

Figure S9. Forest plots per subgroup analysis in studies at low risk of bias. *(continued)*

C. Pneumonia Infection origin

Study	Total	Delirium	Proportion	95%−CI	Weight
**Not Specified**					
Aliberti 2015	172	53	0.31	[0.24; 0.38]	9.2%
Callea 2022	71	20	0.28	[0.18; 0.40]	8.9%
Gholi 2022	310	217	0.70	[0.65; 0.75]	9.3%
Helms 2020	140	118	0.84	[0.77; 0.90]	9.0%
Mendes 2021	235	48	0.20	[0.15; 0.26]	9.3%
Premkumar 2019	110	67	0.61	[0.51; 0.70]	9.2%
Ticinesi 2020	852	94	0.11	[0.09; 0.13]	9.4%
**Random effects model**	**1890**		**0.42**	**[0.17; 0.71]**	**64.3%**
Heterogeneity: $I^2 = 98.7\%$, $\tau^2 = 1.7633$, $p < 0.0001$					
**Community Acquired (CAP)**					
D'Ardes 2021	56	14	0.25	[0.14; 0.38]	8.8%
Pieralli 2014	443	110	0.25	[0.21; 0.29]	9.4%
Riquelme 1997	101	45	0.45	[0.35; 0.55]	9.1%
**Random effects model**	**600**		**0.31**	**[0.11; 0.62]**	**27.3%**
Heterogeneity: $I^2 = 87.1\%$, $\tau^2 = 0.2253$, $p = 0.0004$					
**Mixed (CAP & HCAP)**					
Guimaraes 2023	27	14	**0.52**	**[0.32; 0.71]**	**8.4%**
**Random effects model**	**2517**		**0.40**	**[0.24; 0.58]**	**100.0%**

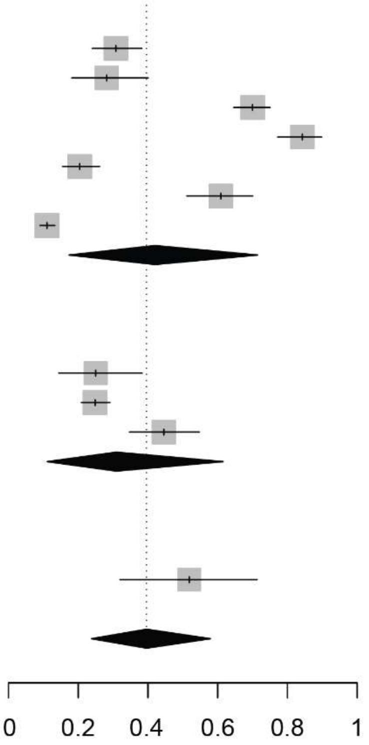

Heterogeneity: $I^2 = 98.0\%$, $\tau^2 = 1.1796$, $p < 0.0001$
Test for subgroup differences: $\chi^2_2 = 3.34$, df = 2 ($p = 0.1885$)

D. Microbiological etiology

Study	Total	Delirium	Proportion	95%−CI	Weight
**COVID-19**					
Callea 2022	71	20	0.28	[0.18; 0.40]	8.9%
D'Ardes 2021	56	14	0.25	[0.14; 0.38]	8.8%
Gholi 2022	310	217	0.70	[0.65; 0.75]	9.3%
Helms 2020	140	118	0.84	[0.77; 0.90]	9.0%
Mendes 2021	235	48	0.20	[0.15; 0.26]	9.3%
Ticinesi 2020	852	94	0.11	[0.09; 0.13]	9.4%
**Random effects model**	**1664**		**0.38**	**[0.12; 0.74]**	**54.7%**
Heterogeneity: $I^2 = 98.9\%$, $\tau^2 = 2.0470$, $p < 0.0001$					
**Bacterial**					
Riquelme 1997	101	45	**0.45**	**[0.35; 0.55]**	**9.1%**
**Viral (non−COVID) & bacterial**					
Aliberti 2015	172	53	0.31	[0.24; 0.38]	9.2%
Premkumar 2019	110	67	0.61	[0.51; 0.70]	9.2%
**Random effects model**	**282**		**0.45**	**[0.00; 1.00]**	**18.4%**
Heterogeneity: $I^2 = 95.8\%$, $\tau^2 = 0.7514$, $p < 0.0001$					
**Not Specified**					
Guimaraes 2023	27	14	0.52	[0.32; 0.71]	8.4%
Pieralli 2014	443	110	0.25	[0.21; 0.29]	9.4%
**Random effects model**	**470**		**0.36**	**[0.00; 1.00]**	**17.8%**
Heterogeneity: $I^2 = 88.5\%$, $\tau^2 = 0.6181$, $p = 0.0032$					
**Random effects model**	**2517**		**0.40**	**[0.24; 0.58]**	**100.0%**

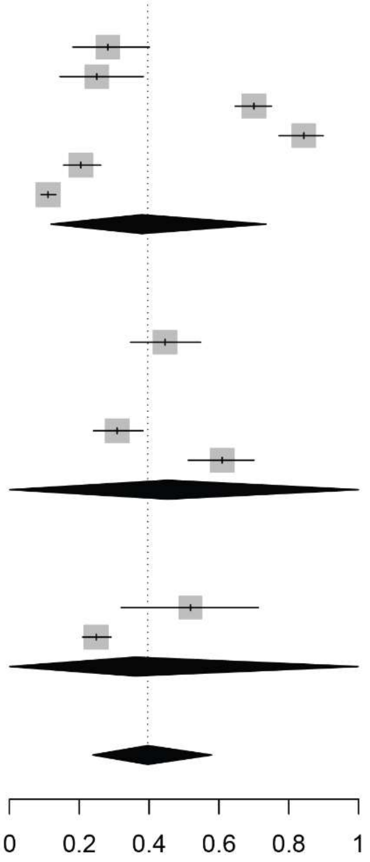

Heterogeneity: $I^2 = 98.0\%$, $\tau^2 = 1.1796$, $p < 0.0001$
Test for subgroup differences: $\chi^2_3 = 0.49$, df = 3 ($p = 0.9201$)

Figure S10. Older age is a predisposing factor for delirium.

A

Study	DOM	[95% CI]	Weight
Yuksel 2021	7.47	[3.32, 11.62]	6.16%
Vinogradov 2021	3.10	[−4.15, 10.35]	4.34%
Tuma 2021	6.20	[−0.87, 13.27]	4.43%
Tomasi 2017	−6.50	[−17.14, 4.14]	2.88%
Ticinesi 2020	9.00	[6.99, 11.01]	7.31%
Morandi 2021	8.20	[5.32, 11.08]	6.89%
Mendes 2021	0.80	[−1.05, 2.65]	7.37%
Martins 2022	6.25	[−0.47, 12.97]	4.62%
Helms 2020	0.33	[−7.71, 8.37]	3.94%
Eggers 2004	1.75	[−7.81, 11.31]	3.27%
D'Ardes 2021	13.31	[7.36, 19.26]	5.06%
Callea 2022	7.33	[1.95, 12.72]	5.40%
Capuzzi 2023	5.20	[0.98, 9.42]	6.12%
Chang 2024	15.30	[13.33, 17.27]	7.32%
Damanti 2023	4.33	[−1.74, 10.40]	5.00%
Gutowski 2023	10.75	[7.18, 14.32]	6.51%
Lee 2023	10.00	[5.49, 14.51]	5.94%
Xing 2020	1.82	[0.16, 3.48]	7.44%
**Random effects model**	**6.48**	**[4.20, 8.75]**	**100%**
		p* < 0.0001	

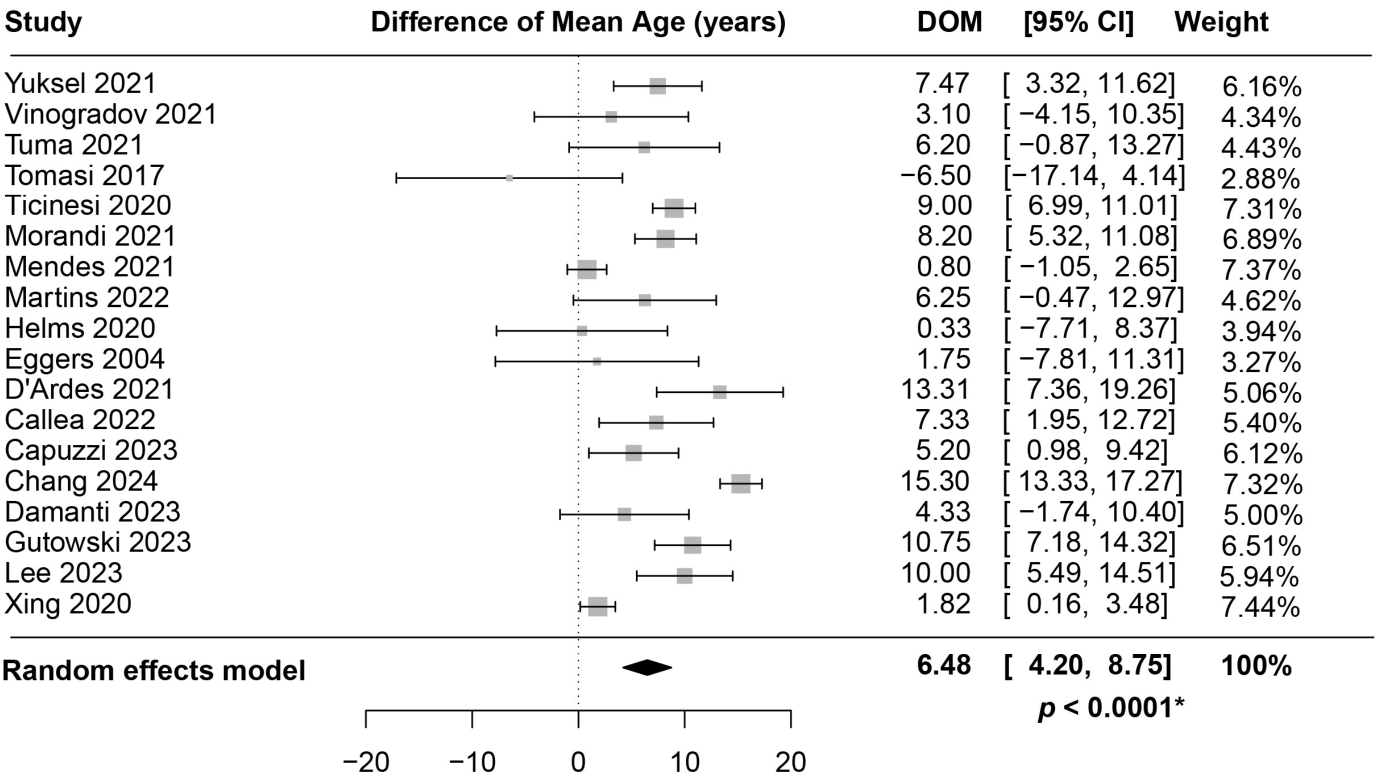

B

Study	Age > 65 Del	Age > 65 Total	Age < 65 Del	Age < 65 Total	OR	95%−CI	Weight
Johnson 2000	36	90	6	58	5.78	[2.25; 14.86]	2.9%
Kelly 2009	16	59	1	21	7.44	[0.92; 60.09]	0.6%
Liu 2007	31	216	4	86	3.44	[1.17; 10.05]	2.2%
Luna 2016	607	3722	169	2472	2.66	[2.22; 3.18]	80.8%
Riquelme 2006	42	109	12	91	4.13	[2.01; 8.47]	5.0%
Ruiz 2014	30	225	9	174	2.82	[1.30; 6.11]	4.3%
Zhang 2018	58	1288	7	614	4.09	[1.86; 9.01]	4.1%
**Random effects model**					**2.87**	**[2.37; 3.47]**	**100.0%**

Heterogeneity: $I^2 = 0\%$, $\tau^2 = 0$ $p = 0.48$ ***p* < 0.0001***

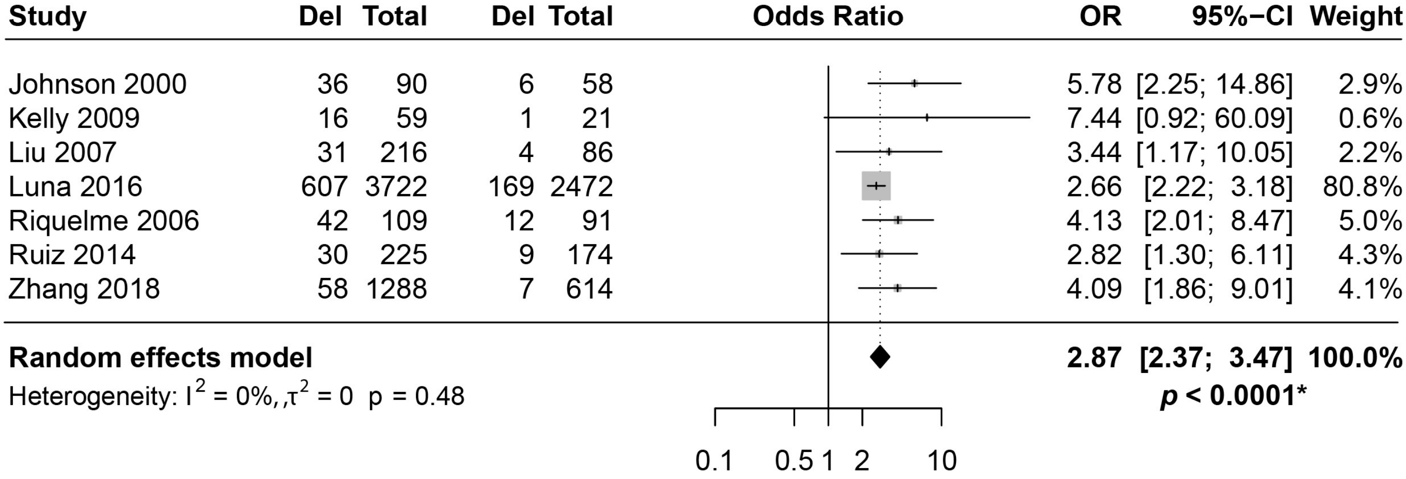

A. Patients with pneumonia and delirium are older than those without delirium (n=18 studies, DOM +6.5 years, 95% CI [4.2; 8.7], *p*<0.0001). **B.** Subgroup analysis by age showed that patients >65 years-old had increased odds of delirium compared to those <65 years old (*n*=7 studies, OR 2.87, 95% CI [2.37;3.47], p<0.0001).

Figure S11. Forest plots by demographics as predisposing factors of delirium

A. Gender (female)

Study	Delirium Events	Delirium Total	No Del Events	No Del Total	OR	95%-CI	Weight
Callea 2022	9	20	21	51	1.17	[0.41; 3.32]	3.5%
Capuzzi 2023	12	54	46	167	0.75	[0.36; 1.55]	5.5%
Chang 2024	109	207	399	931	1.48	[1.10; 2.01]	9.9%
D'Ardes 2021	9	14	28	42	0.90	[0.25; 3.20]	2.6%
Damanti 2023	5	13	23	37	0.38	[0.10; 1.40]	2.5%
Eggers 2004	2	11	1	9	1.78	[0.13; 23.52]	0.8%
Garcia 2021	58	224	315	816	0.56	[0.40; 0.77]	9.5%
Gutowski 2023	42	72	99	263	2.32	[1.36; 3.94]	7.3%
Helms 2020	29	118	11	22	0.33	[0.13; 0.83]	4.1%
Lee 2023	11	26	41	85	0.79	[0.32; 1.91]	4.4%
Martins 2022	11	29	9	30	1.43	[0.48; 4.21]	3.3%
Mendes 2021	24	48	109	187	0.72	[0.38; 1.35]	6.3%
Morandi 2021	21	39	77	202	1.89	[0.95; 3.78]	5.8%
Portela-Sanchez 2021	3	13	18	58	0.67	[0.16; 2.72]	2.2%
Tasci 2022	33	95	22	59	0.90	[0.46; 1.76]	5.9%
Ticinesi 2020	42	94	360	758	0.89	[0.58; 1.37]	8.4%
Tomasi 2017	4	10	13	20	0.36	[0.08; 1.71]	1.9%
Tuma 2021	14	43	6	12	0.48	[0.13; 1.77]	2.5%
Vinogradov 2021	3	10	11	20	0.35	[0.07; 1.76]	1.8%
Xing 2020	12	22	40	78	1.14	[0.44; 2.95]	4.0%
Yuksel 2021	92	213	33	94	1.41	[0.85; 2.32]	7.6%
**Random effects model**		**1375**		**3941**	**0.93**	**[0.73; 1.20]**	**100.0%**

Heterogeneity: $I^2 = 59\%$, $\tau^2 = 0.1202$, $p < 0.01$

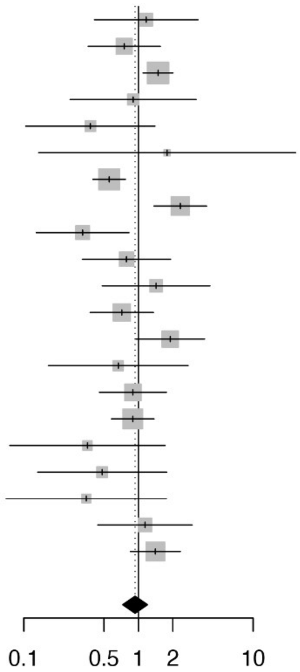

***p* = 0.57**

B. Nursing Home

Study	Delirium Events	Delirium Total	NoDel Events	NoDel Total	OR	95%-CI	Weight
Chang 2024	141	207	178	931	9.04	[6.47; 12.63]	50.5%
Damanti 2023	1	13	1	37	3.00	[0.17; 51.75]	8.6%
Mendes 2021	16	48	33	187	2.33	[1.15; 4.74]	40.9%
**Random effects model**		**268**		**1155**	**4.73**	**[0.64; 35.04]**	**100.0%**

Heterogeneity: $I^2 = 83\%$, $\tau^2 = 0.4001$, $p < 0.01$

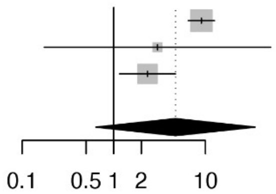

***p* = 0.08**

C. Smoking

Study	Delirium Events	Delirium Total	NoDel Events	NoDel Total	OR	95%-CI	Weight
Garcia-Grimshaw 2022	21	166	129	851	0.81	[0.49; 1.33]	45.4%
Gutowski 2023	5	72	22	263	0.82	[0.30; 2.24]	10.9%
Lee 2023	7	26	27	85	0.79	[0.30; 2.11]	11.6%
Morandi 2021	8	39	53	202	0.73	[0.31; 1.68]	15.8%
Tuma 2021	10	43	2	12	1.52	[0.28; 8.09]	4.0%
Xing 2020	11	22	42	78	0.86	[0.33; 2.21]	12.4%
**Random effects model**		**368**		**1491**	**0.82**	**[0.70; 0.96]**	**100.0%**

Heterogeneity: $I^2 = 0\%$, $\tau^2 = 0$, $p = 0.99$

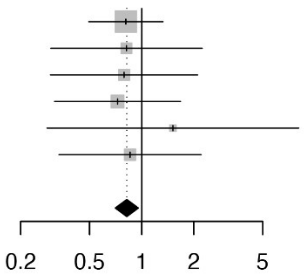

p* = 0.02

D. Alcohol intake

Study	Delirium Events	Delirium Total	NoDel Events	NoDel Total	OR	95%-CI	Weight
Jolley 2023	23	273	221	5706	2.28	[1.46; 3.57]	80.3%
Tuma 2021	4	43	0	12	2.85	[0.14; 56.65]	1.8%
Xing 2020	12	22	38	78	1.26	[0.49; 3.26]	17.9%
**Random effects model**		**338**		**5796**	**2.06**	**[1.02; 4.16]**	**100.0%**

Heterogeneity: $I^2 = 0\%$, $\tau^2 = 0$, $p = 0.53$

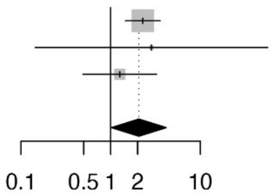

p* = 0.047

Figure S12. Forest plots by comorbidities as predisposing factors of delirium

A. Dementia

Study	Delirium Events	Delirium Total	NoDel Events	NoDel Total	OR	95%−CI	Weight
Bianchetti 2020	55	55	27	27	2.02	[0.04; 104.45]	0.4%
Callea 2022	9	20	4	51	9.61	[2.50; 37.02]	3.6%
Chang 2024	119	207	205	931	4.79	[3.49; 6.57]	32.8%
Garcia−Grimshaw 2022	3	166	11	851	1.41	[0.39; 5.09]	3.9%
Gutowski 2023	6	72	9	263	2.57	[0.88; 7.46]	5.6%
Lee 2023	3	26	2	85	5.41	[0.85; 34.35]	2.0%
Martins 2022	5	29	5	30	1.04	[0.27; 4.06]	3.6%
Mendes 2021	37	48	82	187	4.31	[2.07; 8.96]	10.8%
Morandi 2021	19	39	23	202	7.39	[3.45; 15.86]	10.0%
Ticinesi 2020	38	94	118	758	3.68	[2.33; 5.81]	21.7%
Tuma 2021	5	43	0	12	3.57	[0.18; 69.23]	0.8%
Xing 2020	7	22	8	78	4.08	[1.28; 12.99]	4.8%
**Random effects model**		**821**		**3475**	**4.13**	**[3.07; 5.55]**	**100.0%**

Heterogeneity: $I^2 = 11\%$, $\tau^2 = 0.0294$, $p = 0.34$

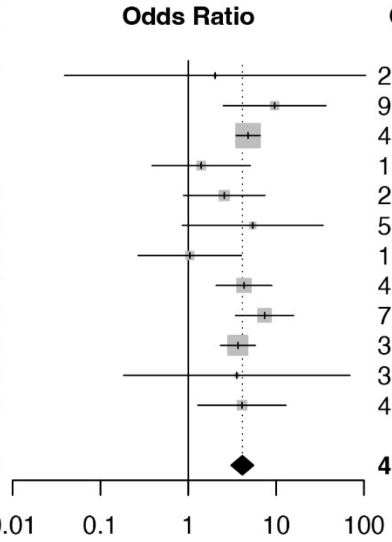

p* <0.001

B. Stroke

Study	Delirium Events	Delirium Total	NoDel Events	NoDel Total	OR	95%−CI	Weight
Callea 2022	7	20	6	51	4.04	[1.15; 14.14]	6.6%
Chang 2024	21	207	65	931	1.50	[0.90; 2.52]	39.0%
Damanti 2023	0	13	4	37	0.28	[0.01; 5.48]	1.2%
Helms 2020	9	118	0	22	3.90	[0.22; 69.53]	1.3%
Lee 2023	2	26	1	85	7.00	[0.61; 80.55]	1.7%
Mendes 2021	13	48	33	187	1.73	[0.83; 3.63]	19.0%
Ticinesi 2020	11	94	43	758	2.20	[1.09; 4.44]	21.2%
Tuma 2021	8	43	0	12	5.99	[0.32; 111.44]	1.2%
Xing 2020	8	22	10	78	3.89	[1.30; 11.59]	8.7%
**Random effects model**		**591**		**2161**	**2.01**	**[1.40; 2.91]**	**100.0%**

Heterogeneity: $I^2 = 0\%$, $\tau^2 = 0$, $p = 0.49$

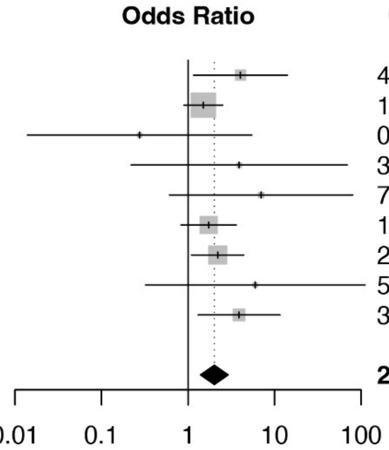

p* = 0.002

C. COPD

Study	Delirium Events	Delirium Total	NoDel Events	NoDel Total	OR	95%−CI	Weight
Callea 2022	3	20	9	51	0.82	[0.20; 3.42]	4.8%
Capuzzi 2023	18	54	32	167	2.11	[1.06; 4.18]	20.8%
Garcia−Grimshaw 2022	8	166	30	851	1.39	[0.62; 3.08]	15.3%
Gutowski 2023	3	72	14	263	0.77	[0.22; 2.77]	6.0%
Helms 2020	20	118	2	22	2.04	[0.44; 9.43]	4.2%
Lee 2023	2	26	4	85	1.69	[0.29; 9.78]	3.2%
Morandi 2021	6	39	21	202	1.57	[0.59; 4.18]	10.1%
Ticinesi 2020	20	94	82	758	2.23	[1.29; 3.84]	32.9%
Tuma 2021	5	43	1	12	1.45	[0.15; 13.72]	1.9%
Vinogradov 2021	1	10	0	20	6.47	[0.24; 174.08]	0.9%
**Random effects model**		**642**		**2431**	**1.75**	**[1.35; 2.27]**	**100.0%**

Heterogeneity: $I^2 = 0\%$, $\tau^2 = 0$, $p = 0.86$

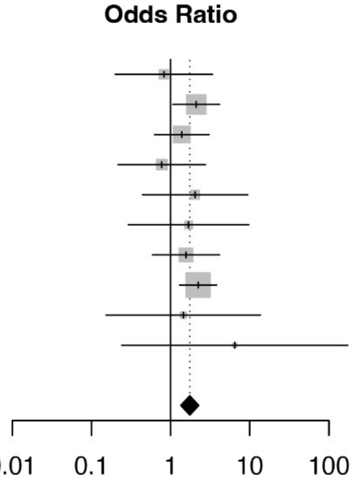

p* = 0.001

Figure S12. Forest plots by comorbidities as predisposing factors of delirium. *(continued)*

D. Kidney disease

Study	Delirium Events	Delirium Total	NoDel Events	NoDel Total	OR	95%-CI	Weight
Callea 2022	4	20	4	51	2.94	[0.66; 13.13]	5.1%
Chang 2024	20	207	74	931	1.24	[0.74; 2.08]	27.6%
Garcia–Grimshaw 2022	5	166	17	851	1.52	[0.55; 4.19]	10.3%
Gutowski 2023	13	72	30	263	1.71	[0.84; 3.48]	18.1%
Helms 2020	6	118	3	22	0.34	[0.08; 1.47]	5.3%
Lee 2023	2	26	2	85	3.46	[0.46; 25.86]	2.9%
Tasci 2022	8	95	0	59	11.56	[0.65; 204.11]	1.5%
Ticinesi 2020	16	94	59	758	2.43	[1.33; 4.43]	23.0%
Tuma 2021	11	43	3	12	1.03	[0.24; 4.51]	5.3%
Vinogradov 2021	0	10	0	20	1.95	[0.04; 105.52]	0.8%
**Random effects model**		**851**		**3052**	**1.62**	**[1.08; 2.43]**	**100.0%**

Heterogeneity: $I^2 = 14\%$, $\tau^2 = 0.0477$, $p = 0.31$

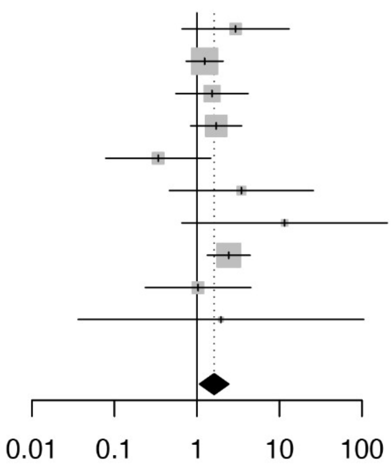

p* <0.026

E. Liver disease

Study	Delirium Events	Delirium Total	NoDel Events	NoDel Total	OR	95%-CI	Weight
Capuzzi 2023	6	54	11	167	1.77	[0.62; 5.05]	46.8%
Chang 2024	4	207	10	931	1.81	[0.56; 5.84]	37.4%
Damanti 2023	0	13	1	37	0.90	[0.03; 23.50]	4.8%
Helms 2020	2	118	0	22	0.97	[0.04; 20.80]	5.4%
Tasci 2022	2	95	0	59	3.18	[0.15; 67.43]	5.5%
**Random effects model**		**487**		**1216**	**1.73**	**[1.23; 2.43]**	**100.0%**

Heterogeneity: $I^2 = 0\%$, $\tau^2 = 0$, $p = 0.98$

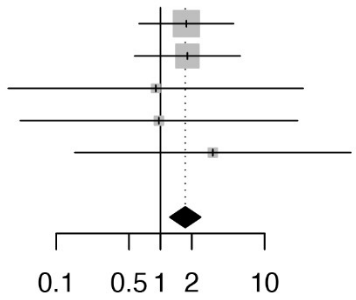

p* = 0.011

F. Hypertension

Study	Delirium Events	Delirium Total	NoDel Events	NoDel Total	OR	95%-CI	Weight
Callea 2022	12	20	25	51	1.56	[0.55; 4.46]	4.9%
Capuzzi 2023	33	54	83	167	1.59	[0.85; 2.97]	9.0%
Chang 2024	66	207	316	931	0.91	[0.66; 1.26]	13.6%
Damanti 2023	11	13	23	37	3.35	[0.65; 17.37]	2.4%
Garcia–Grimshaw 2022	54	166	250	851	1.16	[0.81; 1.66]	13.1%
Gutowski 2023	44	72	148	263	1.22	[0.72; 2.08]	10.3%
Lee 2023	21	26	34	85	6.30	[2.17; 18.32]	4.8%
Mendes 2021	31	48	137	187	0.67	[0.34; 1.31]	8.4%
Morandi 2021	25	39	118	202	1.27	[0.62; 2.59]	7.9%
Tasci 2022	54	95	25	59	1.79	[0.93; 3.45]	8.6%
Ticinesi 2020	65	94	433	758	1.68	[1.06; 2.67]	11.4%
Tuma 2021	31	43	8	12	1.29	[0.33; 5.10]	3.2%
Vinogradov 2021	7	10	8	20	3.50	[0.69; 17.71]	2.5%
**Random effects model**		**887**		**3623**	**1.41**	**[1.04; 1.91]**	**100.0%**

Heterogeneity: $I^2 = 47\%$, $\tau^2 = 0.1175$, $p = 0.03$

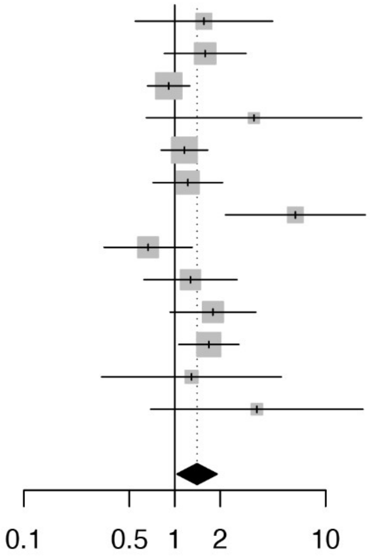

p* = 0.032

Figure S12. Forest plots by comorbidities as predisposing factors of delirium. *(continued)*

G. Heart disease

Study	Delirium Events	Delirium Total	NoDel Events	NoDel Total	OR	95%−CI	Weight
Callea 2022	10	20	19	51	1.68	[0.59; 4.79]	6.0%
Capuzzi 2023	29	54	67	167	1.73	[0.93; 3.21]	8.8%
Chang 2024	13	207	64	931	0.91	[0.49; 1.68]	8.8%
Damanti 2023	4	13	5	37	2.84	[0.63; 12.86]	3.9%
Garcia−Grimshaw 2022	10	166	41	851	1.27	[0.62; 2.58]	8.1%
Gutowski 2023	18	72	36	263	2.10	[1.11; 3.98]	8.7%
Helms 2020	58	118	12	22	0.81	[0.32; 2.01]	6.8%
Lee 2023	7	26	10	85	2.76	[0.93; 8.21]	5.8%
Mendes 2021	24	48	154	187	0.21	[0.11; 0.42]	8.4%
Morandi 2021	18	39	75	202	1.45	[0.73; 2.90]	8.3%
Tasci 2022	65	95	28	59	2.40	[1.23; 4.69]	8.4%
Ticinesi 2020	32	94	204	758	1.40	[0.89; 2.21]	10.0%
Tuma 2021	8	43	0	12	5.99	[0.32; 111.44]	1.4%
Xing 2020	12	22	40	78	1.14	[0.44; 2.95]	6.6%
**Random effects model**		**1017**		**3703**	**1.33**	**[0.89; 1.99]**	**100.0%**

Heterogeneity: $I^2 = 68\%$, $\tau^2 = 0.2964$, $p < 0.01$

***p* = 0.15**

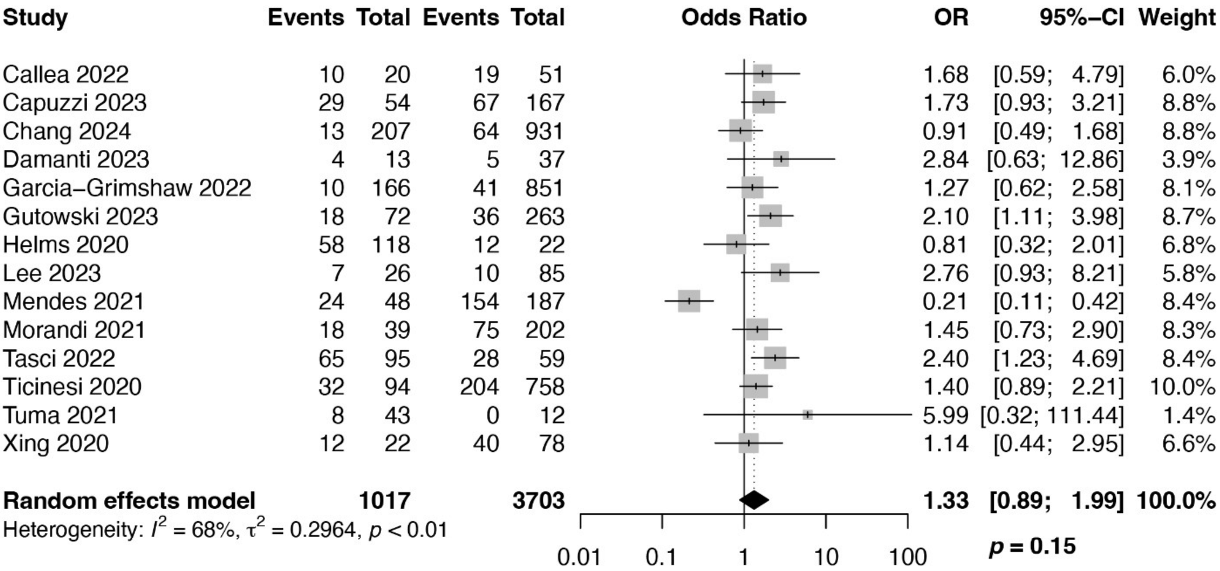

H. Diabetes

Study	Delirium Events	Delirium Total	NoDel Events	NoDel Total	OR	95%−CI	Weight
Callea 2022	7	20	14	51	1.42	[0.47; 4.30]	3.2%
Capuzzi 2023	11	54	37	167	0.90	[0.42; 1.91]	6.9%
Damanti 2023	2	13	11	37	0.43	[0.08; 2.27]	1.4%
Garcia−Grimshaw 2022	45	166	233	851	0.99	[0.68; 1.43]	28.4%
Gutowski 2023	22	72	63	263	1.40	[0.79; 2.48]	12.0%
Helms 2020	18	118	3	22	1.14	[0.31; 4.25]	2.3%
Lee 2023	7	26	22	85	1.06	[0.39; 2.85]	4.0%
Mendes 2021	9	48	45	187	0.73	[0.33; 1.62]	6.2%
Morandi 2021	14	39	43	202	2.07	[0.99; 4.32]	7.3%
Tasci 2022	28	95	11	59	1.82	[0.83; 4.02]	6.4%
Ticinesi 2020	23	94	156	758	1.25	[0.76; 2.07]	15.8%
Tuma 2021	19	43	4	12	1.58	[0.41; 6.06]	2.2%
Vinogradov 2021	2	10	3	20	1.42	[0.20; 10.23]	1.0%
Xing 2020	5	22	10	78	2.00	[0.60; 6.63]	2.8%
**Random effects model**		**820**		**2792**	**1.19**	**[0.99; 1.43]**	**100.0%**

Heterogeneity: $I^2 = 0\%$, $\tau^2 = 0$, $p = 0.76$

***p* = 0.06**

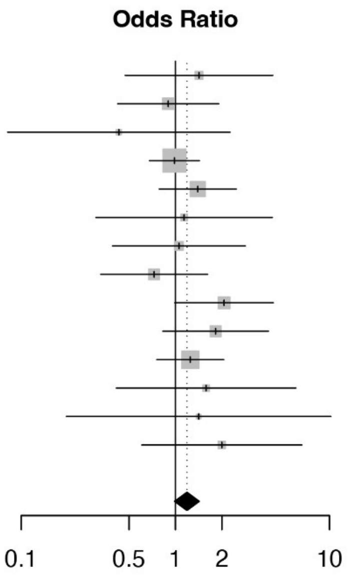

i. Cancer

Study	Delirium Events	Delirium Total	NoDel Events	NoDel Total	OR	95%−CI	Weight
Callea 2022	6	20	5	51	3.94	[1.04; 14.89]	10.4%
Capuzzi 2023	12	54	19	167	2.23	[1.00; 4.95]	15.9%
Chang 2024	14	207	43	931	1.50	[0.80; 2.79]	18.0%
Damanti 2023	0	13	10	37	0.10	[0.01; 1.78]	3.4%
Gutowski 2023	7	72	37	263	0.66	[0.28; 1.54]	15.2%
Helms 2020	16	118	5	22	0.53	[0.17; 1.65]	12.3%
Ticinesi 2020	11	94	119	758	0.71	[0.37; 1.38]	17.6%
Tuma 2021	5	43	2	12	0.66	[0.11; 3.91]	7.3%
**Random effects model**		**621**		**2241**	**1.03**	**[0.51; 2.09]**	**100.0%**

Heterogeneity: $I^2 = 54\%$, $\tau^2 = 0.3910$, $p = 0.03$

***p* = 0.91**

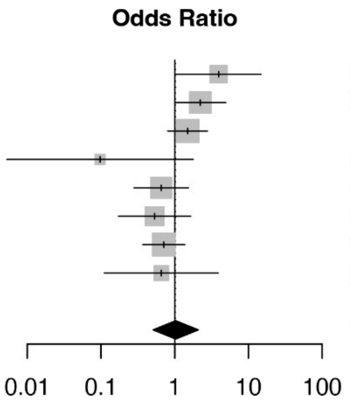

Figure S13. Forest plots by demographics as predisposing factors of delirium in studies at low risk of bias

A. Gender (Female)

Study	Delirium Events	Delirium Total	No Del Events	No Del Total	OR	95%−CI	Weight
Callea 2022	9	20	21	51	1.17	[0.41; 3.32]	10.2%
D'Ardes 2021	9	14	28	42	0.90	[0.25; 3.20]	7.0%
Helms 2020	29	118	11	22	0.33	[0.13; 0.83]	12.5%
Mendes 2021	24	48	109	187	0.72	[0.38; 1.35]	24.7%
Ticinesi 2020	42	94	360	758	0.89	[0.58; 1.37]	45.6%
**Random effects model**		**294**		**1060**	**0.77**	**[0.47; 1.25]**	**100.0%**
						***p* = 0.20**	

Heterogeneity: I^2 = 9.6%, τ^2 = 0.0190, p = 0.3516

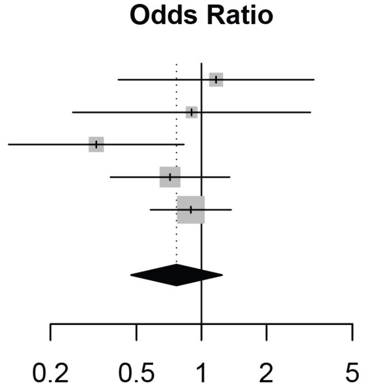

B. Age difference

Study	DOM	95% CI	Weight
Ticinesi 2020	9.00	[6.99, 11.01]	24.14%
Mendes 2021	0.80	[−1.05, 2.65]	24.30%
Helms 2020	0.33	[−7.71, 8.37]	14.56%
D'Ardes 2021	13.31	[7.36, 19.26]	18.01%
Callea 2022	7.33	[1.95, 12.72]	18.99%
**Random−Effects Model**	**6.20**	**[1.49, 10.92]**	**100%**
		p* = 0.01	

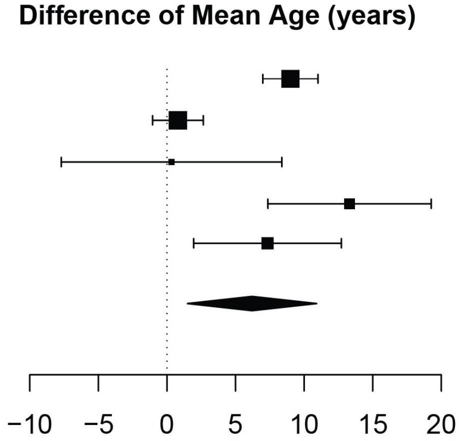

C. Nursing home

Study	Delirium Events	Delirium Total	No Del Events	No Del Total	OR	95%−CI
Mendes 2021	16	48	33	187	2.33	[1.15; 4.74]
						p* = 0.02

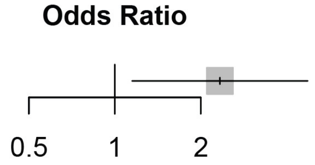

Figure S14. Forest plots by comorbidities as predisposing factors of delirium in studies at low risk of bias

A. Dementia

Study	Delirium Events	Delirium Total	No Del Events	No Del Total	OR	95%−CI	Weight
Callea 2022	9	20	4	51	9.61	[2.50; 37.02]	7.6%
Mendes 2021	37	48	82	187	4.31	[2.07; 8.96]	25.8%
Ticinesi 2020	38	94	118	758	3.68	[2.33; 5.81]	66.5%
**Random effects model**		**162**		**996**	**4.12**	**[1.91; 8.89]**	**100.0%**

p* = 0.015

Heterogeneity: $I^2 = 0.0\%$, $\tau^2 = 0$, $p = 0.4136$

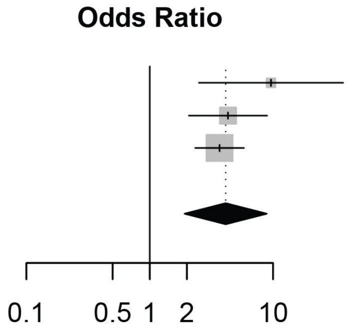

B. Stroke

Study	Delirium Events	Delirium Total	No Del Events	No Del Total	OR	95%−CI	Weight
Callea 2022	7	20	6	51	4.04	[1.15; 14.14]	13.8%
Helms 2020	9	118	0	22	3.90	[0.22; 69.53]	2.6%
Mendes 2021	13	48	33	187	1.73	[0.83; 3.63]	39.5%
Ticinesi 2020	11	94	43	758	2.20	[1.09; 4.44]	44.1%
**Random effects model**		**280**		**1018**	**2.21**	**[1.31; 3.74]**	**100.0%**

p* = 0.017

Heterogeneity: $I^2 = 0.0\%$, $\tau^2 = 0$, $p = 0.6929$

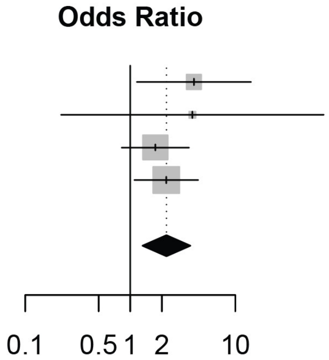

C. COPD

Study	Delirium Events	Delirium Total	No Del Events	No Del Total	OR	95%−CI	Weight
Callea 2022	3	20	9	51	0.82	[0.20; 3.42]	11.5%
Helms 2020	20	118	2	22	2.04	[0.44; 9.43]	9.9%
Ticinesi 2020	20	94	82	758	2.23	[1.29; 3.84]	78.6%
**Random effects model**		**232**		**831**	**1.97**	**[0.75; 5.14]**	**100.0%**

***p* = 0.093**

Heterogeneity: $I^2 = 0.0\%$, $\tau^2 = 0$, $p = 0.4401$

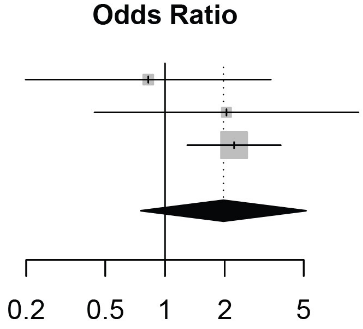

D. Kidney disease

Study	Delirium Events	Delirium Total	No Del Events	No Del Total	OR	95%−CI	Weight
Callea 2022	4	20	4	51	2.94	[0.66; 13.13]	28.6%
Helms 2020	6	118	3	22	0.34	[0.08; 1.47]	29.1%
Ticinesi 2020	16	94	59	758	2.43	[1.33; 4.43]	42.3%
**Random effects model**		**232**		**831**	**1.45**	**[0.08; 24.67]**	**100.0%**

***p* = 0.631**

Heterogeneity: $I^2 = 68.0\%$, $\tau^2 = 0.9330$, $p = 0.0439$

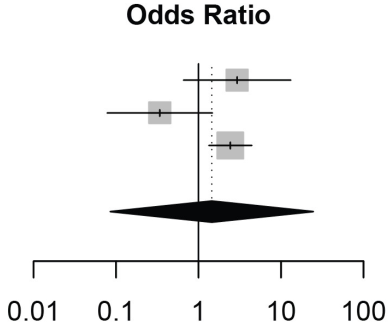

Figure S14. Forest plots by comorbidities as predisposing factors of delirium in studies at low risk of bias. *(continued)*

E. Hypertension

Study	Delirium Events	Delirium Total	No Del Events	No Del Total	OR	95%−CI	Weight
Callea 2022	12	20	25	51	1.56	[0.55; 4.46]	21.1%
Mendes 2021	31	48	137	187	0.67	[0.34; 1.31]	34.2%
Ticinesi 2020	65	94	433	758	1.68	[1.06; 2.67]	44.7%
**Random effects model**		**162**		**996**	**1.21**	**[0.33; 4.45]**	**100.0%**
						***p* = 0.60**	

Heterogeneity: $I^2 = 60.8\%$, $\tau^2 = 0.1511$, $p = 0.0780$

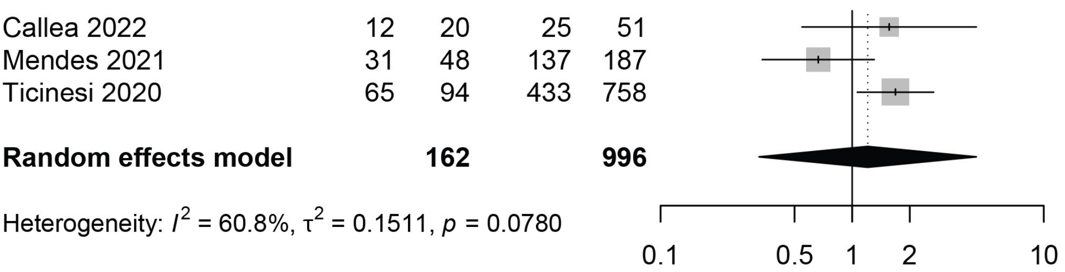

F. Heart disease

Study	Delirium Events	Delirium Total	No Del Events	No Del Total	OR	95%−CI	Weight
Callea 2022	10	20	19	51	1.68	[0.59; 4.79]	21.9%
Helms 2020	58	118	12	22	0.81	[0.32; 2.01]	23.5%
Mendes 2021	24	48	154	187	0.21	[0.11; 0.42]	26.2%
Ticinesi 2020	32	94	204	758	1.40	[0.89; 2.21]	28.4%
**Random effects model**		**280**		**1018**	**0.78**	**[0.18; 3.51]**	**100.0%**
						***p* = 0.64**	

Heterogeneity: $I^2 = 86.4\%$, $\tau^2 = 0.7262$, $p < 0.0001$

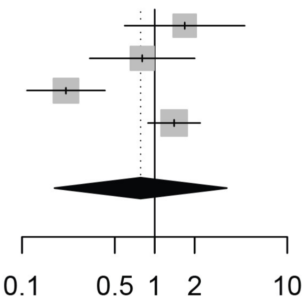

G. Diabetes

Study	Delirium Events	Delirium Total	No Del Events	No Del Total	OR	95%−CI	Weight
Callea 2022	7	20	14	51	1.42	[0.47; 4.30]	11.8%
Helms 2020	18	118	3	22	1.14	[0.31; 4.25]	8.3%
Mendes 2021	9	48	45	187	0.73	[0.33; 1.62]	22.6%
Ticinesi 2020	23	94	156	758	1.25	[0.76; 2.07]	57.2%
**Random effects model**		**280**		**1018**	**1.11**	**[0.72; 1.72]**	**100.0%**
						***p* = 0.48**	

Heterogeneity: $I^2 = 0.0\%$, $\tau^2 = 0$, $p = 0.6867$

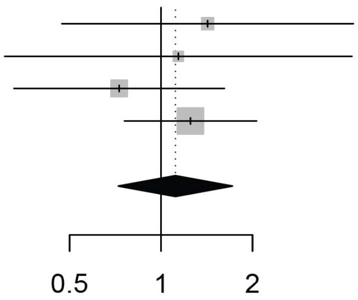

H. Cancer

Study	Delirium Events	Delirium Total	No Del Events	No Del Total	OR	95%−CI	Weight
Callea 2022	6	20	5	51	3.94	[1.04; 14.89]	28.5%
Helms 2020	16	118	5	22	0.53	[0.17; 1.65]	31.8%
Ticinesi 2020	11	94	119	758	0.71	[0.37; 1.38]	39.6%
**Random effects model**		**232**		**831**	**1.06**	**[0.08; 13.62]**	**100.0%**
						***p* = 0.93**	

Heterogeneity: $I^2 = 66.9\%$, $\tau^2 = 0.7766$, $p = 0.0486$

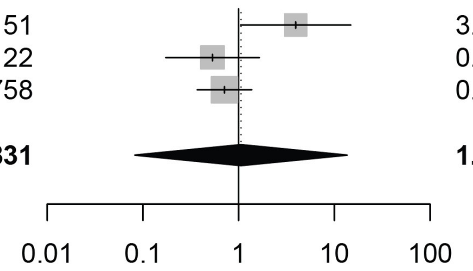

Figure S15. Forest plots by pneumonia-severity factors

A. ICU Admission

Study	Delirium Events	Delirium Total	NoDel Events	NoDel Total	OR	95%−CI	Weight
Carr 2019	73	73	29	29	2.49	[0.05; 128.51]	1.8%
Cooper 2020	11	11	16	16	0.70	[0.01; 37.73]	1.8%
Denke 2018	37	37	5	5	6.82	[0.12; 380.08]	1.8%
Eggers 2004	11	11	9	9	1.21	[0.02; 66.96]	1.8%
Ekmekyapar 2022	64	64	486	486	0.13	[0.00; 6.74]	1.9%
Gholi 2022	217	217	93	93	2.33	[0.05; 118.11]	1.9%
GomezDuque 2023	148	148	135	135	1.10	[0.02; 55.61]	1.9%
Gutowski 2023	8	72	63	263	0.40	[0.18; 0.87]	19.1%
Helms 2020	118	118	22	22	5.27	[0.10; 272.37]	1.8%
Iribarren−Diarasarri 2023	63	63	80	80	0.79	[0.02; 40.31]	1.8%
Kaneko 2022	24	24	88	88	0.28	[0.01; 14.31]	1.8%
Lee 2023	26	26	85	85	0.31	[0.01; 16.00]	1.8%
Limpawattana 2016	21	21	15	15	1.39	[0.03; 73.79]	1.8%
Lin 2008	14	14	35	35	0.41	[0.01; 21.58]	1.8%
Martins 2022	29	29	30	30	0.97	[0.02; 50.36]	1.8%
Portela−Sanchez 2021	3	13	10	58	1.44	[0.33; 6.19]	9.8%
Premkumar 2019	67	67	43	43	1.55	[0.03; 79.66]	1.8%
Regueiro−Mira 2015	6	17	33	223	3.14	[1.09; 9.08]	14.5%
Tomasi 2017	4	10	0	20	28.38	[1.34; 600.58]	3.0%
vanderKnaap 2024	48	48	276	276	0.18	[0.00; 8.95]	1.8%
Yuksel 2021	163	213	32	94	6.32	[3.71; 10.75]	24.3%
**Random effects model**		**1296**		**2105**	**1.73**	**[0.96; 3.10]**	**100.0%**

Heterogeneity: $I^2 = 55.0\%$, $\tau^2 = 0.2518$, $p = 0.0013$

***p* = 0.07**

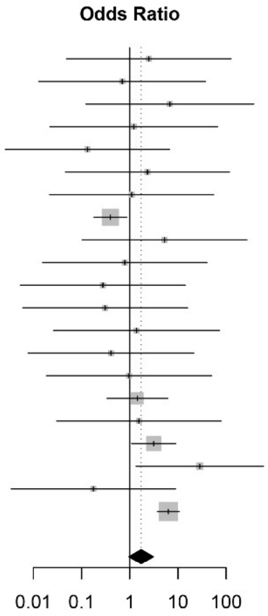

B. Invasive Ventilation

Study	Delirium Events	Delirium Total	NoDel Events	NoDel Total	OR	95%−CI	Weight
Carr 2019	73	73	29	29	2.49	[0.05; 128.51]	3.3%
Damanti 2023	0	13	0	37	2.78	[0.05; 147.03]	3.2%
Eggers 2004	11	11	9	9	1.21	[0.02; 66.96]	3.2%
Garcia−Grimshaw 2022	131	166	116	851	23.72	[15.56; 36.14]	14.2%
GomezDuque 2023	148	148	135	135	1.10	[0.02; 55.61]	3.3%
Helms 2020	118	118	22	22	5.27	[0.10; 272.37]	3.3%
Iribarren−Diarasarri 2023	63	63	80	80	0.79	[0.02; 40.31]	3.3%
Kaneko 2022	24	24	88	88	0.28	[0.01; 14.31]	3.3%
Lee 2023	22	26	10	85	41.25	[11.78; 144.43]	10.9%
Lin 2008	14	14	35	35	0.41	[0.01; 21.58]	3.2%
Martins 2022	26	29	18	30	5.78	[1.42; 23.44]	10.2%
Tasci 2022	38	95	18	59	1.52	[0.76; 3.03]	13.4%
Tuma 2021	19	43	5	12	1.11	[0.30; 4.05]	10.7%
vanderKnaap 2024	48	48	276	276	0.18	[0.00; 8.95]	3.3%
Yuksel 2021	52	213	3	94	9.80	[2.98; 32.26]	11.2%
**Random effects model**		**1084**		**1842**	**3.86**	**[1.60; 9.33]**	**100.0%**

Heterogeneity: $I^2 = 81\%$, $\tau^2 = 1.1436$, $p < 0.01$

p* = 0.005

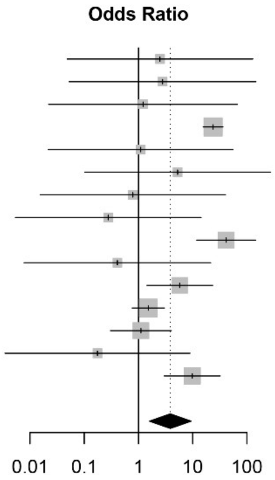

C. Noninvasive Ventilation

Study	Delirium Events	Delirium Total	NoDel Events	NoDel Total	OR	95%−CI	Weight
Callea 2022	9	20	9	51	3.82	[1.22; 11.91]	8.9%
Carr 2019	0	73	0	29	0.40	[0.01; 20.70]	2.9%
Damanti 2023	0	13	4	37	0.28	[0.01; 5.48]	4.2%
Eggers 2004	0	11	0	9	0.83	[0.01; 45.69]	2.8%
Garcia−Grimshaw 2022	35	166	735	851	0.04	[0.03; 0.06]	10.6%
GomezDuque 2023	0	148	0	135	0.91	[0.02; 46.30]	2.9%
Helms 2020	0	118	0	22	0.19	[0.00; 9.82]	2.9%
Iribarren−Diarasarri 2023	0	63	0	80	1.27	[0.02; 64.78]	2.9%
Kaneko 2022	0	24	0	88	3.61	[0.07; 186.74]	2.9%
Lee 2023	4	26	75	85	0.02	[0.01; 0.08]	8.5%
Lin 2008	0	14	0	35	2.45	[0.05; 129.36]	2.9%
Martins 2022	3	29	12	30	0.17	[0.04; 0.70]	8.1%
Morandi 2021	5	39	25	202	1.04	[0.37; 2.91]	9.2%
Tasci 2022	57	95	41	59	0.66	[0.33; 1.31]	10.1%
Tuma 2021	24	43	7	12	0.90	[0.25; 3.30]	8.4%
vanderKnaap 2024	0	48	0	276	5.70	[0.11; 290.74]	2.9%
Yuksel 2021	161	213	91	94	0.10	[0.03; 0.34]	8.7%
**Random effects model**		**1143**		**2095**	**0.39**	**[0.17; 0.92]**	**100.0%**

Heterogeneity: $I^2 = 87\%$, $\tau^2 = 1.4799$, $p < 0.01$

p* = 0.03

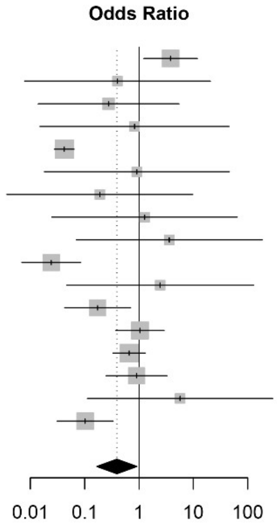

Figure S15. Forest plots by pneumonia-severity factors. *(continued)*

D. Multilobar pneumonia

Study	Delirium Events	Delirium Total	NoDel Events	NoDel Total	OR	95%-CI	Weight
Garcia–Grimshaw 2022	129	166	425	851	3.49	[2.37; 5.16]	26.0%
Morandi 2021	10	39	64	202	0.74	[0.34; 1.62]	17.0%
Portela–Sanchez 2021	11	13	45	58	1.59	[0.31; 8.09]	6.6%
Trevisan 2023	158	197	717	963	1.39	[0.95; 2.03]	26.2%
Vinogradov 2021	10	10	20	20	0.51	[0.01; 27.68]	1.3%
Yuksel 2021	153	213	62	94	1.32	[0.78; 2.22]	22.8%
**Random effects model**		**638**		**2188**	**1.56**	**[0.84; 2.90]**	**100.0%**

Heterogeneity: $I^2 = 75\%$, $\tau^2 = 0.1829$, $p < 0.01$

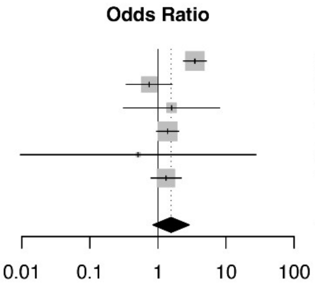

***p* = 0.12**

E. Dialysis

Study	Delirium Events	Delirium Total	NoDel Events	NoDel Total	OR	95%-CI	Weight
Garcia–Grimshaw 2022	43	166	64	851	4.30	[2.79; 6.61]	89.2%
Lee 2023	3	26	2	85	5.41	[0.85; 34.35]	4.8%
Tuma 2021	11	43	2	12	1.72	[0.32; 9.09]	6.0%
**Random effects model**		**235**		**948**	**4.12**	**[2.07; 8.17]**	**100.0%**

Heterogeneity: $I^2 = 0\%$, $\tau^2 = 0$, $p = 0.55$

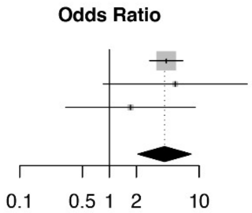

p* = 0.01

F. Steroids

Study	Delirium Events	Delirium Total	NoDel Events	NoDel Total	OR	95%-CI	Weight
Callea 2022	20	20	49	51	2.07	[0.10; 45.04]	10.1%
Chang 2024	33	207	199	931	0.70	[0.47; 1.04]	55.3%
Damanti 2023	3	13	27	37	0.11	[0.03; 0.49]	28.0%
Lee 2023	26	26	85	85	0.31	[0.01; 16.00]	6.6%
**Random effects model**		**266**		**1104**	**0.44**	**[0.08; 2.52]**	**100.0%**

Heterogeneity: $I^2 = 52\%$, $\tau^2 = 0.4985$, $p = 0.10$

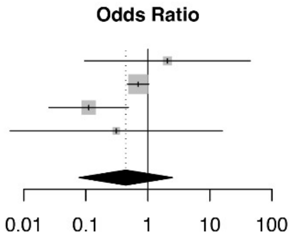

***p* = 0.23**

Figure S16. Forest plots by pneumonia-severity factors in studies at low risk of bias.

A. ICU Admision

Study	Delirium Events	Delirium Total	No Del Events	No Del Total	OR	95%−CI	Weight
Gholi 2022	217	217	93	93	2.33	[0.05; 118.11]	33.5%
Helms 2020	118	118	22	22	5.27	[0.10; 272.37]	33.2%
Premkumar 2019	67	67	43	43	1.55	[0.03; 79.66]	33.3%
**Random effects model**		**402**		**158**	**2.67**	**[0.57; 12.49]**	**100.0%**

Heterogeneity: $I^2 = 0.0\%$, $\tau^2 = 0$, $p = 0.9087$

$p = 0.11$

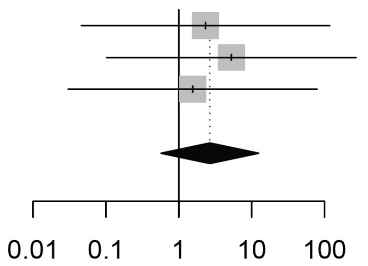

B. Invasive ventilation

Study	Delirium Events	Delirium Total	No Del Events	No Del Total	OR	95%−CI
Helms 2020	118	118	22	22	5.27	[0.10; 272.37]

$p = 0.41$

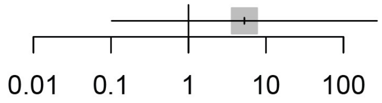

C. Steroids

Study	Delirium Events	Delirium Total	No Del Events	No Del Total	OR	95%−CI
Callea 2022	20	20	49	51	2.07	[0.10; 45.04]

$p = 0.63$

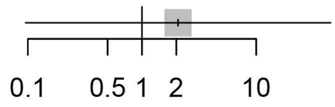

Figure S17. Forest plots of the associations between delirium and clinical course

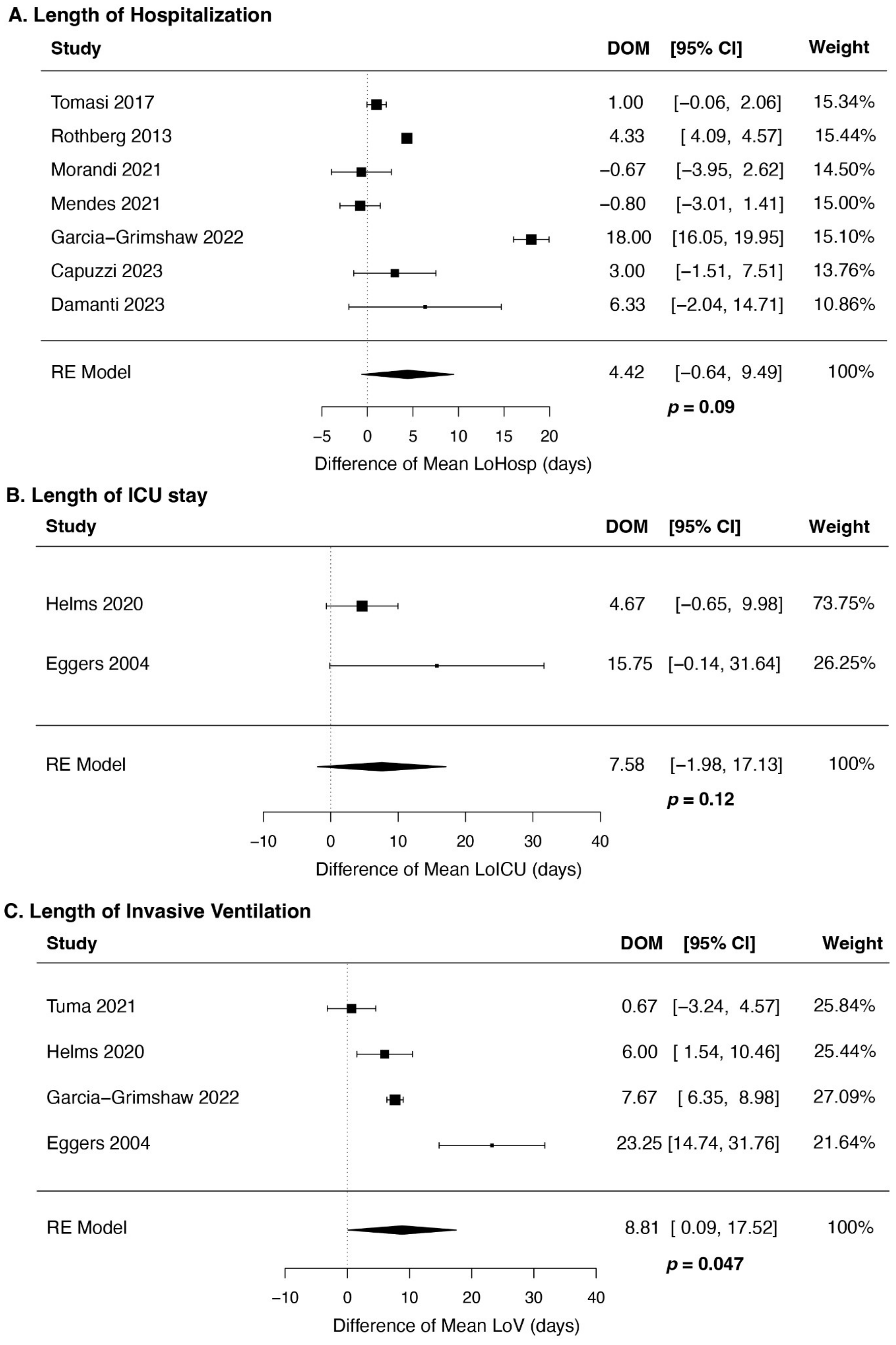

A. Patients with pneumonia and delirium did not show significant differences in hospitalization length or in ICU stay (**B**), but they did show increased length of invasive ventilation (**C**, DOM 8.81, 95% CI [0.09, 17.52], *p*=0.047). Difference of means (DOM) was calculated using the Hozo/Wan/Bland method.

Figure S18. Forest plots of the associations between delirium and clinical course in studies at low risk of bias

A. Length of Hospitalization

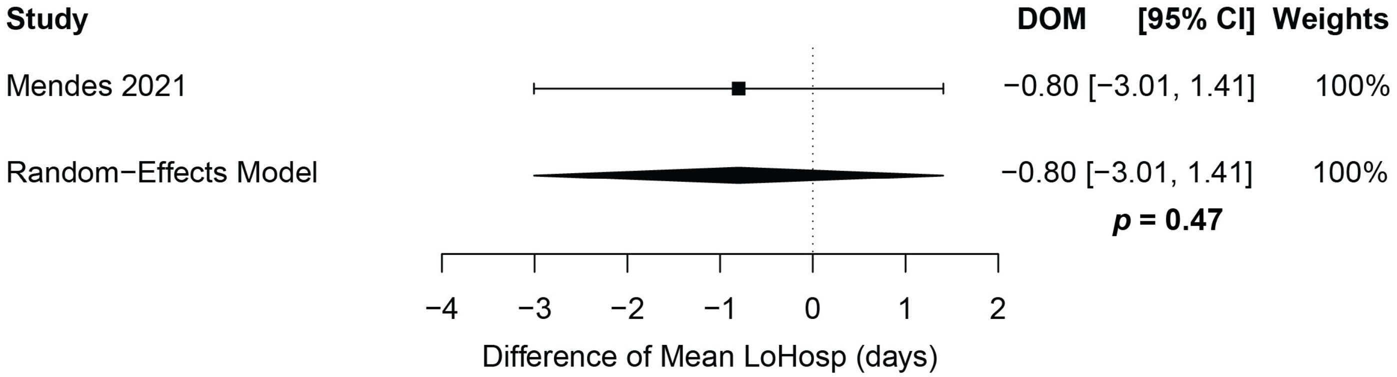

B. Length of ICU stay

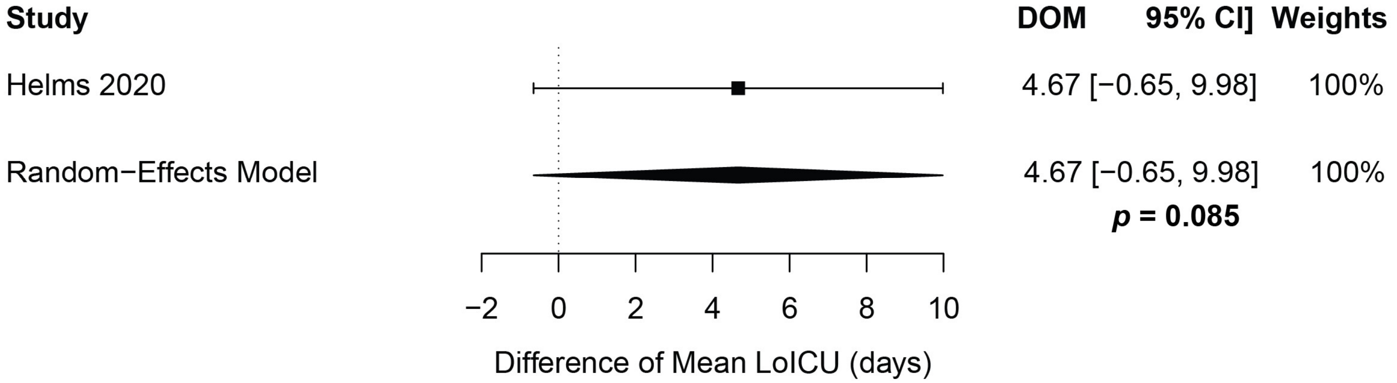

C. Length of Invasive Ventilation

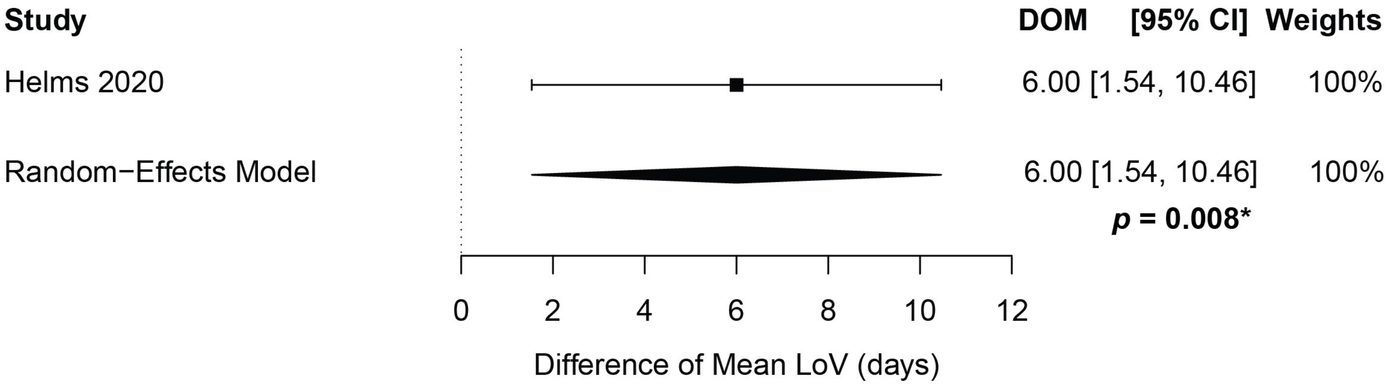

Figure S19. Delirium is associated with significantly increased mortality in patients with pneumonia in studies at low risk of bias.

A. Overall death univariate

Study	Delirium Events	Delirium Total	No Del Events	No Del Total	OR	95%−CI	Weight
Aliberti 2015	45	53	22	119	24.80	[10.26; 59.98]	17.1%
Callea 2022	7	20	3	51	8.62	[1.95; 38.03]	13.2%
Helms 2020	19	118	2	22	1.92	[0.41; 8.90]	12.9%
Mendes 2021	20	48	56	187	1.67	[0.87; 3.21]	18.5%
Pieralli 2014	17	110	37	333	1.46	[0.79; 2.72]	18.7%
Ticinesi 2020	54	94	227	758	3.16	[2.04; 4.89]	19.6%
**Random effects model**		**443**		**1470**	**3.70**	**[1.14; 12.05]**	**100.0%**

Heterogeneity: $I^2 = 84.6\%$, $\tau^2 = 1.0258$, $p < 0.0001$

$p = 0.036$*

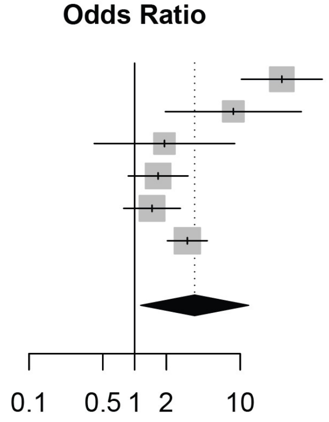

B. Overall death multivariate

Study	logOR	SE(logOR)	OR	95%−CI	Weight
Aliberti 2015	0.8544	0.3742	2.35	[1.13; 4.90]	25.3%
Callea 2022	1.9592	0.9898	7.09	[1.02; 49.38]	3.6%
Mendes 2021	0.7419	0.2873	2.10	[1.20; 3.70]	43.0%
Pieralli 2014	1.7387	0.7237	5.69	[1.36; 23.20]	6.8%
Ticinesi 2020	0.2078	0.4076	1.23	[0.55; 2.74]	21.3%
**Random effects model (HK)**			**2.16**	**[1.19; 3.91]**	**100.0%**

Heterogeneity: $I^2 = 23.0\%$, $\tau^2 < 0.0001$, $p = 0.2676$

$p = 0.023$*

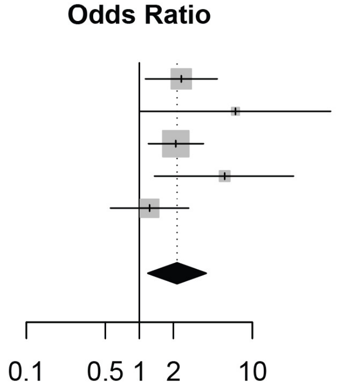

Supplementary References

1. Charlson ME, Pompei P, Ales KL, MacKenzie CR. A new method of classifying prognostic comorbidity in longitudinal studies: development and validation. *J Chronic Dis*. 1987;40(5):373-383. doi:10.1016/0021-9681(87)90171-8

2. Lim WS, van der Eerden MM, Laing R, et al. Defining community acquired pneumonia severity on presentation to hospital: an international derivation and validation study. *Thorax*. 2003;58(5):377-382. doi:10.1136/thorax.58.5.377

3. Fine MJ, Auble TE, Yealy DM, et al. A prediction rule to identify low-risk patients with community-acquired pneumonia. *N Engl J Med*. 1997;336(4):243-250. doi:10.1056/NEJM199701233360402

4. *Diagnostic and Statistical Manual of Mental Disorders: DSM-5™, 5th Ed*. American Psychiatric Publishing, Inc.; 2013:xliv, 947. doi:10.1176/appi.books.9780890425596

5. Inouye SK, van Dyck CH, Alessi CA, Balkin S, Siegal AP, Horwitz RI. Clarifying confusion: the confusion assessment method. A new method for detection of delirium. *Ann Intern Med*. 1990;113(12):941-948. doi:10.7326/0003-4819-113-12-941

6. Bergeron N, Dubois MJ, Dumont M, Dial S, Skrobik Y. Intensive Care Delirium Screening Checklist: evaluation of a new screening tool. *Intensive Care Med*. 2001;27(5):859-864. doi:10.1007/s001340100909

7. Trzepacz PT, Baker RW, Greenhouse J. A symptom rating scale for delirium. *Psychiatry Res*. 1988;23(1):89-97. doi:10.1016/0165-1781(88)90037-6

8. Gaudreau JD, Gagnon P, Harel F, Tremblay A, Roy MA. Fast, systematic, and continuous delirium assessment in hospitalized patients: the nursing delirium screening scale. *J Pain Symptom Manage*. 2005;29(4):368-375. doi:10.1016/j.jpainsymman.2004.07.009

9. Schuurmans MJ, Shortridge-Baggett LM, Duursma SA. The Delirium Observation Screening Scale: a screening instrument for delirium. *Res Theory Nurs Pract*. 2003;17(1):31-50. doi:10.1891/rtnp.17.1.31.53169

10. Inouye SK, Leo-Summers L, Zhang Y, Bogardus Jr. ST, Leslie DL, Agostini JV. A Chart-Based Method for Identification of Delirium: Validation Compared with Interviewer Ratings Using the Confusion Assessment Method. *Journal of the American Geriatrics Society*. 2005;53(2):312-318. doi:10.1111/j.1532-5415.2005.53120.x

11. Sessler CN, Gosnell MS, Grap MJ, et al. The Richmond Agitation–Sedation Scale. *Am J Respir Crit Care Med*. 2002;166(10):1338-1344. doi:10.1164/rccm.2107138

12. Teasdale G, Jennett B. Assessment of coma and impaired consciousness. A practical scale. *Lancet*. 1974;2(7872):81-84. doi:10.1016/s0140-6736(74)91639-0

13. HODKINSON HM. EVALUATION OF A MENTAL TEST SCORE FOR ASSESSMENT OF MENTAL IMPAIRMENT IN THE ELDERLY. *Age and Ageing*. 1972;1(4):233-238. doi:10.1093/ageing/1.4.233

14. Ferenci P, Lockwood A, Mullen K, Tarter R, Weissenborn K, Blei AT. Hepatic encephalopathy--definition, nomenclature, diagnosis, and quantification: final report of the working party at the 11th World Congresses of Gastroenterology, Vienna, 1998. *Hepatology*. 2002;35(3):716-721. doi:10.1053/jhep.2002.31250

15. Aromataris E, Lockwood C, Porritt K, Pilla B, Jordan Z. *JBI Manual for Evidence Synthesis.* JBI https://synthesismanual.jbi.global. https://doi.org/10.46658/JBIMES-24-01

16. Aliberti S, Bellelli G, Belotti M, et al. Delirium symptoms during hospitalization predict long-term mortality in patients with severe pneumonia. *Aging Clin Exp Res*. 2015;27(4):523-531. doi:10.1007/s40520-014-0297-9

17. Aliyu ZY, Aliyu MH, McCormick K. Determinants for hospitalization in " low-risk" community acquired pneumonia. *BMC Infectious Diseases*. 2003;3:11. doi:10.1186/1471-2334-3-11

18. Andrea BR, Benchimol-Barbosa PR, Farah S, Monteiro A. Clinical and Laboratory Data Up on Hospital Admission are Predictors of New-Onset Atrial Fibrillation in Patients Hospitalized Due to COVID-19 Pneumonia. *Arq Bras Cardiol*. 2024;121(1):e20220784. doi:10.36660/abc.20220784

19. Aziz KT, Best MJ, Naseer Z, et al. The Association of Delirium with Perioperative Complications in Primary Elective Total Hip Arthroplasty. *Clin Orthop Surg*. 2018;10(3):286-291. doi:10.4055/cios.2018.10.3.286

20. Beretta S, Cristillo V, Camera G, et al. Incidence and Long-term Functional Outcome of Neurologic Disorders in Hospitalized Patients With COVID-19 Infected With Pre-Omicron Variants. *Neurology*. 2023;101(9):e892-e903. doi:10.1212/WNL.0000000000207534

21. Bhansali S, Bagrodia V, Choudhury S, et al. Spectrum of hospitalized NeuroCOVID diagnoses from a tertiary care neurology centre in Eastern India. *Journal of Clinical Neuroscience*. 2021;93:96-102. doi:10.1016/j.jocn.2021.09.008

22. Bianchetti A, Rozzini R, Guerini F, et al. Clinical Presentation of COVID19 in Dementia Patients. *The Journal of Nutrition, Health & Aging*. 2020;24(6):560. doi:10.1007/s12603-020-1389-1

23. Blagoeva V, Hodzhev V, Dimova R, Stoyanova R, Bahariev D. Predictors of a severe course and mortality in patients with COVID-19-associated pneumonia. *Folia Med (Plovdiv)*. 2024;66(1):59-65. doi:10.3897/folmed.66.e111124

24. Blot M, Croisier D, Péchinot A, et al. A leukocyte score to improve clinical outcome predictions in bacteremic pneumococcal pneumonia in adults. *Open Forum Infect Dis*. 2014;1(2):ofu075. doi:10.1093/ofid/ofu075

25. Callan KT, Donnelly M, Lung B, et al. Risk factors for postoperative delirium in orthopaedic hip surgery patients: a database review. *BMC Musculoskelet Disord*. 2024;25(1):71. doi:10.1186/s12891-024-07174-x

26. Callea A, Conti G, Fossati B, et al. Delirium in hospitalized patients with COVID-19 pneumonia: a prospective, cross-sectional, cohort study. *Internal and Emergency Medicine*. 2022;17(5):1445. doi:10.1007/s11739-022-02934-w

27. Capuzzi E, Caldiroli A, Cella F, Turco M, Buoli M, Clerici M. Sociodemographic and clinical characteristics associated with delirium in hospitalized patients with COVID-19: are immigrants a vulnerable group. *Intern Emerg Med*. 2023;18(3):937-941. doi:10.1007/s11739-023-03232-9

28. Carr C, Smith A, Marturano M, et al. Ventilator-Associated Pneumonia: How Do the Different Criteria for Diagnosis Match Up? *The American Surgeon™*. 2019;85(9):992-997. doi:10.1177/000313481908500941

29. Cataneo-Piña DJ, Hernández-Favela CG, Mondragón-Posadas LA, Torres Nuñez C. Geriatric care-related outcomes in patients 75 years and older admitted to a pulmonary disease center and predictors for hospital-related complications. *Aging Med (Milton)*. 2023;6(4):353-360. doi:10.1002/agm2.12271

30. Ceriani E, Pitino A, Radovanovic D, et al. Continuous Positive Airway Pressure in Elderly Patients with Severe COVID-19 Related Respiratory Failure. *J Clin Med*. 2022;11(15):4454. doi:10.3390/jcm11154454

31. Chang SC, Grunkemeier GL, Goldman JD, et al. A simplified pneumonia severity index (PSI) for clinical outcome prediction in COVID-19. *PLoS One*. 2024;19(5):e0303899. doi:10.1371/journal.pone.0303899

32. Chen T, Wu D, Chen H, et al. Clinical characteristics of 113 deceased patients with coronavirus disease 2019: retrospective study. *BMJ*. 2020;368:m1091. doi:10.1136/bmj.m1091

33. Clemente MG, Budiño TG, Seco GA, Santiago M, Gutiérrez M, Romero P. [Community-acquired pneumonia in the elderly: prognostic factors]. *Arch Bronconeumol*. 2002;38(2):67-71. doi:10.1016/s0300-2896(02)75154-5

34. Cooper J, Stukas S, Hoiland RL, et al. Quantification of Neurological Blood-Based Biomarkers in Critically Ill Patients With Coronavirus Disease 2019. *Crit Care Explor*. 2020;2(10):e0238. doi:10.1097/CCE.0000000000000238

35. D'Ardes D, Carrarini C, Russo M, et al. Low molecular weight heparin in COVID-19 patients prevents delirium and shortens hospitalization. *Neurol Sci*. 2021;42(4):1527-1530. doi:10.1007/s10072-020-04887-4

36. Damanti S, Cilla M, Vitali G, et al. Exploring the Association between Delirium and Malnutrition in COVID-19 Survivors: A Geriatric Perspective. *Nutrients*. 2023;15(22):4727. doi:10.3390/nu15224727

37. Haan E de, Rijckevorsel VAJIM van, Bod P, Roukema GR, Jong L de, Collaboration (DHFR) O behalf of DHFR. Delirium After Surgery for Proximal Femoral Fractures in the Frail Elderly Patient: Risk Factors and Clinical Outcomes. *Clinical Interventions in Aging*. 2023;18:193. doi:10.2147/CIA.S390906

38. Denke C, Balzer F, Menk M, et al. Long-term sequelae of acute respiratory distress syndrome caused by severe community-acquired pneumonia: Delirium-associated cognitive impairment and post-traumatic stress disorder. *J Int Med Res*. 2018;46(6):2265-2283. doi:10.1177/0300060518762040

39. Díaz Fuenzalida A, Vera C, Santamarina J, et al. [Community-acquired pneumonia in the elderly requiring hospitalization. Clinical features and prognosis]. *Medicina (B Aires)*. 1999;59(6):731-738.

40. Dravid A, Kashiva R, Khan Z, et al. Combination therapy of Tocilizumab and steroid for management of COVID-19 associated cytokine release syndrome: A single center experience from Pune, Western India. *Medicine*. 2021;100(29):e26705. doi:10.1097/MD.0000000000026705

41. Eggers V, Fügener K, Hein OV, et al. Antibiotic-mediated release of tumour necrosis factor alpha and norharman in patients with hospital-acquired pneumonia and septic encephalopathy. *Intensive Care Med*. 2004;30(8):1544-1551. doi:10.1007/s00134-004-2285-6

42. Ekmekyapar T, Ekmekyapar M, Tasci I, Sahin L, Delen LA. Clinical features and predisposing factors of delirium due to COVID-19 pneumonia in intensive care units. *Eur Rev Med Pharmacol Sci*. 2022;26(12):4440-4448. doi:10.26355/eurrev_202206_29083

43. Fernández-Sabé N, Carratalà J, Rosón B, et al. Community-acquired pneumonia in very elderly patients: causative organisms, clinical characteristics, and outcomes. *Medicine (Baltimore)*. 2003;82(3):159-169. doi:10.1097/01.md.0000076005.64510.87

44. Fimognari FL, Corsonello A, Rizzo M, et al. Contribution of clinical severity and geriatric risk factors in predicting short-term mortality of older hospitalized pneumonia patients: the Pneumonia in Italian Acute Care for Elderly units (PIACE) study. *Aging Clin Exp Res*. 2022;34(6):1419-1427. doi:10.1007/s40520-021-02063-y

45. García S, Cuatepotzo-Burgos FM, Toledo-Lozano CG, et al. Neurological Manifestations and Outcomes in a Retrospective Cohort of Mexican Inpatients with SARS-CoV-2 Pneumonia: Design of a Risk Profile. *Healthcare (Basel)*. 2021;9(11):1501. doi:10.3390/healthcare9111501

46. García-Grimshaw M, Chiquete E, Jiménez-Ruiz A, et al. Delirium and Associated Factors in a Cohort of Hospitalized Patients With Coronavirus Disease 2019. *J Acad Consult Liaison Psychiatry*. 2022;63(1):3-13. doi:10.1016/j.jaclp.2021.06.008

47. Garcia-Vidal C, Fernández-Sabé N, Carratalà J, et al. Early mortality in patients with community-acquired pneumonia: causes and risk factors. *Eur Respir J*. 2008;32(3):733-739. doi:10.1183/09031936.00128107

48. Ghaffari M, Ansari H, Beladimoghadam N, et al. Neurological features and outcome in COVID-19: dementia can predict severe disease. *J Neurovirol*. 2021;27(1):86-93. doi:10.1007/s13365-020-00918-0

49. Gholi Z, Yadegarynia D, Eini-Zinab H, Vahdat Shariatpanahi Z. Vitamin D deficiency is Associated with Increased Risk of Delirium and Mortality among Critically Ill, Elderly Covid-19 Patients. *Complement Ther Med*. 2022;70:102855. doi:10.1016/j.ctim.2022.102855

50. Gil D R, Undurraga P A, Saldías P F, Jiménez P P, Barros M M. [Prognostic factors and outcome of community-acquired pneumonia in hospitalized adult patients]. *Rev Med Chil*. 2006;134(11):1357-1366. doi:10.4067/s0034-98872006001100002

51. Gogol M, Schmidt D, Dettmer-Flügge A, Vaske B. [Pneumonia in the elderly: results of quality improvement program for a geriatric department in Lower Saxony 2006-2009]. *Z Gerontol Geriatr*. 2011;44(4):235-239. doi:10.1007/s00391-011-0217-y

52. Gómez Duque M, Medina R, Enciso C, et al. Usefulness of Inhaled Sedation in Patients With Severe ARDS Due to COVID-19. *Respir Care*. 2023;68(3):293-299. doi:10.4187/respcare.10371

53. Gonçalves NG, Aliberti MJR, Bertola L, et al. Dissipating the fog: Cognitive trajectories and risk factors 1 year after COVID-19 hospitalization. *Alzheimers Dement*. 2023;19(9):3771-3782. doi:10.1002/alz.12993

54. Goss CH, Rubenfeld GD, Park DR, Sherbin VL, Goodman MS, Root RK. Cost and incidence of social comorbidities in low-risk patients with community-acquired pneumonia admitted to a public hospital. *Chest*. 2003;124(6):2148-2155. doi:10.1378/chest.124.6.2148

55. Guimarães L, Piedade J, Duarte J, et al. Hepatic Encephalopathy in Cirrhotic Patients With Bacterial Infections: Frequency, Clinical Characteristics, and Prognostic Relevance. *J Clin Exp Hepatol*. 2023;13(4):559-567. doi:10.1016/j.jceh.2023.01.004

56. Gupta AB, Flanders SA, Petty LA, et al. Inappropriate Diagnosis of Pneumonia Among Hospitalized Adults. *JAMA Intern Med*. 2024;184(5):548-556. doi:10.1001/jamainternmed.2024.0077

57. Gutowski M, Klimkiewicz J, Michałowski A, Ordak M, Możański M, Lubas A. ICU Delirium Is Associated with Cardiovascular Burden and Higher Mortality in Patients with Severe COVID-19 Pneumonia. *Journal of Clinical Medicine*. 2023;12(15):5049. doi:10.3390/jcm12155049

58. Hai CN, Duc TB, Minh TN, et al. Predicting mortality risk in hospitalized COVID-19 patients: an early model utilizing clinical symptoms. *BMC Pulm Med*. 2024;24(1):24. doi:10.1186/s12890-023-02838-1

59. Helms CM, Viner JP, Sturm RH, Renner ED, Johnson W. Comparative features of pneumococcal, mycoplasmal, and Legionnaires' disease pneumonias. *Ann Intern Med*. 1979;90(4):543-547. doi:10.7326/0003-4819-90-4-543

60. Helms J, Kremer S, Merdji H, et al. Delirium and encephalopathy in severe COVID-19: a cohort analysis of ICU patients. *Critical Care*. 2020;24(1):491. doi:10.1186/s13054-020-03200-1

61. Hoogewerf M, Oosterheert JJ, Hak E, Hoepelman IM, Bonten MJM. Prognostic factors for early clinical failure in patients with severe community-acquired pneumonia. *Clin Microbiol Infect*. 2006;12(11):1097-1104. doi:10.1111/j.1469-0691.2006.01535.x

62. Hwang JH, Handigund M, Hwang JH, Cho YG, Kim DS, Lee J. Clinical Features and Risk Factors Associated With 30-Day Mortality in Patients With Pneumonia Caused by Hypervirulent Klebsiella pneumoniae (hvKP). *Ann Lab Med*. 2020;40(6):481-487. doi:10.3343/alm.2020.40.6.481

63. Iribarren-Diarasarri S, Bermúdez-Ampudia C, Barreira-Mendez R, et al. Post-intensive care syndrome one month after discharge in surviving critically ill COVID-19 patients. *Med Intensiva (Engl Ed)*. 2023;47(9):493-500. doi:10.1016/j.medine.2022.10.012

64. Johnson JC, Jayadevappa R, Baccash PD, Taylor L. Nonspecific presentation of pneumonia in hospitalized older people: age effect or dementia? *J Am Geriatr Soc*. 2000;48(10):1316-1320. doi:10.1111/j.1532-5415.2000.tb02607.x

65. Jolley SE, Mowry CJ, Erlandson KM, Wilson MP, Burnham EL. Impact of Alcohol Misuse on Requirements for Critical Care Services and Development of Hospital Delirium in Patients With COVID-19 pneumonia. *Crit Care Explor*. 2023;5(1):e0829. doi:10.1097/CCE.0000000000000829

66. Jones J. Risk and outcome of aspiration pneumonia in a city hospital. *J Natl Med Assoc*. 1993;85(7):533-536.

67. KANEKO K, ISHIZAKA M, CHIBA K, et al. Characteristics of Patients with Severe COVID-19 Pneumonia Who Could Walk Independently at the Time of Discharge. *Rigaku ryoho kagaku*. 2022;37(6):537-542. doi:10.1589/rika.37.537

68. Kelly E, MacRedmond RE, Cullen G, Greene CM, McElvaney NG, O'Neill SJ. Community-acquired pneumonia in older patients: does age influence systemic cytokine levels in community-acquired pneumonia? *Respirology*. 2009;14(2):210-216. doi:10.1111/j.1440-1843.2008.01423.x

69. Kolditz M, Ewig S, Klapdor B, et al. Community-acquired pneumonia as medical emergency: predictors of early deterioration. *Thorax*. 2015;70(6):551-558. doi:10.1136/thoraxjnl-2014-206744

70. Laurichesse H, Gerbaud L, Baud O, Gourdon F, Beytout J, Study Group of General Practitioners. Hospitalization decision for ambulatory patients with community-acquired pneumonia: a prospective study with general practitioners in France. *Infection*. 2001;29(6):320-325. doi:10.1007/s15010-001-1020-2

71. Lee JH, Han WH, Chun JY, Choi YJ, Han MR, Kim JH. Delirium in patients with COVID-19 treated in the intensive care unit. *PLoS One*. 2023;18(11):e0289662. doi:10.1371/journal.pone.0289662

72. Leijte WT, Wagemaker NMM, van Kraaij TDA, et al. [Mortality and re-admission after hospitalization with COVID-19]. *Ned Tijdschr Geneeskd*. 2020;164:D5423.

73. Lima BR de, Nunes BKG, Guimarães LC da C, Almeida LF de, Pagotto V. Incidence of delirium following hospitalization of elderly people with fractures: risk factors and mortality. *Rev Esc Enferm USP*. 2021;55:e20200467. doi:10.1590/1980-220X-REEUSP-2020-0467

74. Limpawattana P, Panitchote A, Tangvoraphonkchai K, et al. Delirium in critical care: a study of incidence, prevalence, and associated factors in the tertiary care hospital of older Thai adults. *Aging Ment Health*. 2016;20(1):74-80. doi:10.1080/13607863.2015.1035695

75. Lin SM, Huang CD, Liu CY, et al. Risk factors for the development of early-onset delirium and the subsequent clinical outcome in mechanically ventilated patients. *J Crit Care*. 2008;23(3):372-379. doi:10.1016/j.jcrc.2006.09.001

76. Lin RY, Heacock LC, Fogel JF. Drug-induced, dementia-associated and non-dementia, non-drug delirium hospitalizations in the United States, 1998-2005: an analysis of the national inpatient sample. *Drugs Aging*. 2010;27(1):51-61. doi:10.2165/11531060-000000000-00000

77. Lin RY, Heacock LC, Bhargave GA, Fogel JF. Clinical associations of delirium in hospitalized adult patients and the role of on admission presentation. *Int J Geriatr Psychiatry*. 2010;25(10):1022-1029. doi:10.1002/gps.2500

78. Liu H, Zhang T tuo, Wu B quan, Huang J, Zhou Y qi, Zhu J xin. [Clinical analysis of community-acquired pneumonia in the elderly]. *Zhonghua Nei Ke Za Zhi*. 2007;46(10):810-814.

79. Liu YH, Wang YR, Wang QH, et al. Post-infection cognitive impairments in a cohort of elderly patients with COVID-19. *Mol Neurodegener*. 2021;16(1):48. doi:10.1186/s13024-021-00469-w

80. Liu R, Liu N, Suo S, et al. Incidence and risk factors of postoperative delirium following hepatic resection: a retrospective national inpatient sample database study. *BMC Surgery*. 2024;24(1):151. doi:10.1186/s12893-024-02436-w

81. Loponen P, Luther M, Wistbacka JO, et al. Postoperative delirium and health related quality of life after coronary artery bypass grafting. *Scand Cardiovasc J*. 2008;42(5):337-344. doi:10.1080/14017430801939217

82. Luna CM, Palma I, Niederman MS, et al. The Impact of Age and Comorbidities on the Mortality of Patients of Different Age Groups Admitted with Community-acquired Pneumonia. *Ann Am Thorac Soc*. 2016;13(9):1519-1526. doi:10.1513/AnnalsATS.201512-848OC

83. Manali ED, Kolilekas L, Petasakis I, et al. The adherence of Greek chest physicians to CAP Guidelines: The role of patient-related factors. *Pneumon*. 24(4):361-367.

84. Marrie TJ, Fine MJ, Kapoor WN, Coley CM, Singer DE, Obrosky DS. Community-acquired pneumonia and do not resuscitate orders. *J Am Geriatr Soc*. 2002;50(2):290-299. doi:10.1046/j.1532-5415.2002.50061.x

85. Marrie TJ, Huang JQ. Community-acquired pneumonia in patients receiving home care. *J Am Geriatr Soc*. 2005;53(5):834-839. doi:10.1111/j.1532-5415.2005.53264.x

86. Marrie TJ, Huang JQ. Admission is not always necessary for patients with community-acquired pneumonia in risk classes IV and V diagnosed in the emergency room. *Canadian Respiratory Journal*. 2007;14(4):212. doi:10.1155/2007/451417

87. Martínez J, Hernández-Gea V, Rodríguez-de-Santiago E, et al. Bacterial infections in patients with acute variceal bleeding in the era of antibiotic prophylaxis. *J Hepatol*. 2021;75(2):342-350. doi:10.1016/j.jhep.2021.03.026

88. Martins S, Ferreira AR, Fernandes J, et al. Delirium in Patients with Severe COVID-19: Preliminary Results of the MAPA Longitudinal Study. *Acta Med Port*. 2022;35(3):228-230. doi:10.20344/amp.17522

89. Matkovska NR, Virstiuk NH, Balan UV. State of endogenous intoxication and immune-inflammatory response in patients with alcoholic liver cirrhosis associated with non-alcoholic fatty liver disease. *GASTRO*. 2021;53(2):91-97. doi:10.22141/2308-2097.53.2.2019.168982

90. Melchio R, Giamello JD, Testa E, et al. RDW-based clinical score to predict long-term survival in community-acquired pneumonia: a European derivation and validation study. *Intern Emerg Med*. 2021;16(6):1547-1557. doi:10.1007/s11739-020-02615-6

91. Mendes A, Herrmann FR, Périvier S, Gold G, Graf CE, Zekry D. Delirium in Older Patients With COVID-19: Prevalence, Risk Factors, and Clinical Relevance. *J Gerontol A Biol Sci Med Sci*. 2021;76(8):e142-e146. doi:10.1093/gerona/glab039

92. Méndez R, Balanzá-Martínez V, Luperdi SC, et al. Short-term neuropsychiatric outcomes and quality of life in COVID-19 survivors. *J Intern Med*. 2021;290(3):621-631. doi:10.1111/joim.13262

93. Morandi A, Rebora P, Isaia G, et al. Delirium symptoms duration and mortality in SARS-COV2 elderly: results of a multicenter retrospective cohort study. *Aging Clin Exp Res*. 2021;33(8):2327-2333. doi:10.1007/s40520-021-01899-8

94. Mortensen EM, Coley CM, Singer DE, et al. Causes of death for patients with community-acquired pneumonia: results from the Pneumonia Patient Outcomes Research Team cohort study. *Arch Intern Med*. 2002;162(9):1059-1064. doi:10.1001/archinte.162.9.1059

95. Otani K, Miura A, Miyai H, Fukushima H, Matsuishi K. Characteristics of COVID-19 delirium intervened by a psychiatric liaison team in the first 2 years of the COVID-19 pandemic in Japan. *Psychiatry Clin Neurosci*. 2022;76(11):599-600. doi:10.1111/pcn.13456

96. Ozlu T, Bülbül Y, Taşbakan S, et al. General Characteristics and Prognostic Factors of Pneumonia Cases Developed During Pandemic (H1N1) Influenza-A Virus Infection in Turkey. *Balkan Med J*. 2013;30(1):68-73. doi:10.5152/balkanmedj.2012.089

97. Peñafiel FS, Tapia AP, Nesvadba DF, et al. [Severity of community-acquired pneumonia due to coronavirus SARS-CoV-2 in immunocompetent hospitalized adult patients]. *Rev Med Chil*. 2023;151(9):1207-1220. doi:10.4067/s0034-98872023000901207

98. Pieralli F, Vannucchi V, Mancini A, et al. Delirium is a predictor of in-hospital mortality in elderly patients with community acquired pneumonia. *Intern Emerg Med*. 2014;9(2):195-200. doi:10.1007/s11739-013-0991-1

99. Portela-Sánchez S, Sánchez-Soblechero A, Melgarejo Otalora PJ, et al. Neurological complications of COVID-19 in hospitalized patients: The registry of a neurology department in the first wave of the pandemic. *Eur J Neurol*. 2021;28(10):3339-3347. doi:10.1111/ene.14748

100. Prabhahar A, Vijaykumar NA, Selvam S, et al. Characteristics and Prognosis of Infectious Disease Emergencies in Patients with Chronic Kidney Disease in India. *Indian J Crit Care Med*. 2024;28(6):601-606. doi:10.5005/jp-journals-10071-24731

101. Premkumar M, Devurgowda D, Dudha S, et al. A/H1N1/09 Influenza is Associated With High Mortality in Liver Cirrhosis. *J Clin Exp Hepatol*. 2019;9(2):162-170. doi:10.1016/j.jceh.2018.04.006

102. Quah J, Liew CJY, Zou L, et al. Chest radiograph-based artificial intelligence predictive model for mortality in community-acquired pneumonia. *BMJ Open Respir Res*. 2021;8(1):e001045. doi:10.1136/bmjresp-2021-001045

103. Regueiro-Mira MV, Pita-Fernández S, Pértega-Díaz S, López-Calviño B, Seoane-Pillado T, Fernández-Albalat-Ruiz M. [Prognostic factors in adult patients hospitalized for pneumonia caused by Legionella pneumophila]. *Rev Chilena Infectol*. 2015;32(4):435-444. doi:10.4067/S0716-10182015000500010

104. Riquelme R, Torres A, el-Ebiary M, et al. Community-acquired pneumonia in the elderly. Clinical and nutritional aspects. *Am J Respir Crit Care Med*. 1997;156(6):1908-1914. doi:10.1164/ajrccm.156.6.9702005

105. Riquelme O R, Riquelme O M, Rioseco Z ML, Gómez M V, Gil D R, Torres M A. [Etiology and prognostics factors of community-acquired pneumonia among adults patients admitted to a regional hospital in Chile]. *Rev Med Chil*. 2006;134(5):597-605. doi:10.4067/s0034-98872006000500008

106. Rothberg MB, Herzig SJ, Pekow PS, Avrunin J, Lagu T, Lindenauer PK. Association between sedating medications and delirium in older inpatients. *J Am Geriatr Soc*. 2013;61(6):923-930. doi:10.1111/jgs.12253

107. Ruiz LA, Zalacain R, Capelastegui A, et al. Bacteremic pneumococcal pneumonia in elderly and very elderly patients: host- and pathogen-related factors, process of care, and outcome. *J Gerontol A Biol Sci Med Sci*. 2014;69(8):1018-1024. doi:10.1093/gerona/glt288

108. Sabzwari S, Kumar D, Bhanji S, Sheerani M, Azhar G. Proportion, Predictors and Outcomes of Delirium at a Tertiary care Hospital in Karachi, Pakistan. *Ageing Int*. 2014;39(1):33-45. doi:10.1007/s12126-012-9152-5

109. Sakakibara R, Iimura A, Ogata T, et al. Brain diseases and aspiration pneumonia in older person. *Neurology and Clinical Neuroscience*. 2022;10(1):9-13. doi:10.1111/ncn3.12564

110. Saldías F, Mardónez JM, Marchesse M, Viviani P, Farías G, Díaz A. [Community-acquired pneumonia in hospitalized adult patients. Clinical presentation and prognostic factors]. *Rev Med Chil*. 2002;130(12):1373-1382.

111. Serrano L, Ruiz LA, Perez-Fernandez S, et al. Short- and long-term prognosis of patients with community-acquired Legionella or pneumococcal pneumonia diagnosed by urinary antigen testing. *Int J Infect Dis*. 2023;134:106-113. doi:10.1016/j.ijid.2023.05.065

112. Serrano Fernández L, Ruiz Iturriaga LA, España Yandiola PP, et al. Bacteraemic pneumococcal pneumonia and SARS-CoV-2 pneumonia: differences and similarities. *Int J Infect Dis*. 2022;115:39-47. doi:10.1016/j.ijid.2021.11.023

113. Shirakawa C, Shiroshita A, Shiba H, et al. The prognostic factors of in-hospital death among patients with pneumonic COPD acute exacerbation. *Respir Investig*. 2022;60(2):271-276. doi:10.1016/j.resinv.2021.11.009

114. Soares R, Fernandes A, Taveira I, Marreiros A, Nzwalo H. Predictors of pneumonia in patients with acute spontaneous intracerebral hemorrhage in Algarve, Southern Portugal. *Clin Neurol Neurosurg*. 2022;221:107387. doi:10.1016/j.clineuro.2022.107387

115. Sorrell L, Leta V, Barnett A, et al. Clinical features and outcomes of hospitalised patients with COVID-19 and Parkinsonian disorders: A multicentre UK-based study. *PLoS One*. 2023;18(7):e0285349. doi:10.1371/journal.pone.0285349

116. Sousa Matias D, Fielli M, González A, Zurita Villarroel I, Fernández A. [Mortality in bacterial pneumonia due to pneumococcus]. *Medicina (B Aires)*. 2024;84(3):481-486.

117. Suwanpasu, S, Pongpaew,W, Praparpak, W. Geriatric resource nurse programme to maintain functional status of elderly patients hospitalised for community-acquired pneumonia. *Asian J Gerontol Geriatr*. 2016;11(1). https://www.ajgg.org/en-ajgg_issue-details-4.html

118. Szylińska A, Rotter I, Listewnik M, et al. Postoperative Delirium in Patients with Chronic Obstructive Pulmonary Disease after Coronary Artery Bypass Grafting. *Medicina (Kaunas)*. 2020;56(7):342. doi:10.3390/medicina56070342

119. Tasci I, Balgetir F, Mungen B, et al. Evaluation of neurological disorders that develop concurrently with COVID-19 pneumonia: a retrospective analysis. *Arquivos de Neuro-Psiquiatria*. 2022;80(4):375. doi:10.1590/0004-282X-ANP-2021-0059

120. Thabet N, Shindo Y, Okumura J, et al. Clinical characteristics and risk factors for mortality in patients with community-acquired staphylococcal pneumonia. *Nagoya J Med Sci*. 2022;84(2):247-259. doi:10.18999/nagjms.84.2.247

121. Ticinesi A, Cerundolo N, Parise A, et al. Delirium in COVID-19: epidemiology and clinical correlations in a large group of patients admitted to an academic hospital. *Aging Clin Exp Res*. 2020;32(10):2159-2166. doi:10.1007/s40520-020-01699-6

122. Tomasi CD, Vuolo F, Generoso J, et al. Biomarkers of Delirium in a Low-Risk Community-Acquired Pneumonia-Induced Sepsis. *Mol Neurobiol*. 2017;54(1):722-726. doi:10.1007/s12035-016-9708-6

123. Trevisan C, Grande G, Rebora P, et al. Early Onset Delirium During Hospitalization Increases In-Hospital and Postdischarge Mortality in COVID-19 Patients: A Multicenter Prospective Study. *J Clin Psychiatry*. 2023;84(5):22m14565. doi:10.4088/JCP.22m14565

124. Tuma RL, Guedes BF, Carra R, et al. Clinical, cerebrospinal fluid, and neuroimaging findings in COVID-19 encephalopathy: a case series. *Neurol Sci*. 2021;42(2):479-489. doi:10.1007/s10072-020-04946-w

125. Uginet M, Breville G, Assal F, et al. COVID-19 encephalopathy: Clinical and neurobiological features. *J Med Virol*. 2021;93(7):4374-4381. doi:10.1002/jmv.26973

126. van der Knaap N, de Vreeze F, van Rosmalen F, et al. The incidence of neurological complications in mechanically ventilated COVID-19 ICU patients: An observational single-center cohort study in three COVID-19 periods. *Clin Neurol Neurosurg*. 2024;241:108311. doi:10.1016/j.clineuro.2024.108311

127. Viasus D, Cordero E, Rodríguez-Baño J, et al. Changes in epidemiology, clinical features and severity of influenza A (H1N1) 2009 pneumonia in the first post-pandemic influenza season. *Clin Microbiol Infect*. 2012;18(3):E55-62. doi:10.1111/j.1469-0691.2011.03753.x

128. Vinogradov OI, Ogarkova TK, Shamtieva KV, et al. Predictors of Acute Encephalopathy in Patients with COVID-19. *J Clin Med*. 2021;10(21):4821. doi:10.3390/jcm10214821

129. Viscogliosi G, Chiriac IM, Braucci S, Terracina D, Andreozzi P, Ettorre E. Predicting Delirium in Older Patients Hospitalized for Community-Acquired Pneumonia. *J Am Med Dir Assoc*. 2016;17(12):1156-1157. doi:10.1016/j.jamda.2016.07.027

130. Watts SH, Bryan ED. Emergency Department Pneumonia Patients Who do not Meet the Six-Hour Criteria for Antibiotic Administration: Do They Have a Different Clinical Presentation? *J Clin Med Res*. 2012;4(5):338-345. doi:10.4021/jocmr1092w

131. Wrenn JO, Christensen MA, Ward MJ. Limitations in the use of automated mental status detection for clinical decision support. *Int J Med Inform*. 2023;180:105247. doi:10.1016/j.ijmedinf.2023.105247

132. Xing, L., Wang, X.X., Chen, Y.J., Gao, J.Y. Serum calcium level and influencing factors of delirium in patients with severe pneumonia. *Chinese Journal of Clinical Research*. 2020;33(11):1495-1497.

133. Yang Q, Wang J, Huang X, Xu Y, Zhang Y. Incidence and risk factors associated with postoperative delirium following primary elective total hip arthroplasty: a retrospective nationwide inpatient sample database study. *BMC Psychiatry*. 2020;20(1):343. doi:10.1186/s12888-020-02742-6

134. Yang Q, Wang J, Chen Y, Lian Q, Shi Z, Zhang Y. Incidence and risk factors of postoperative delirium following total knee arthroplasty: A retrospective Nationwide Inpatient Sample database study. *Knee*. 2022;35:61-70. doi:10.1016/j.knee.2022.02.006

135. Yang Q, Fu J, Pan X, et al. A retrospective analysis of the incidence of postoperative delirium and the importance of database selection for its definition. *BMC Psychiatry*. 2023;23(1):88. doi:10.1186/s12888-023-04576-4

136. Yavuz, B.G., Çolak,S., Guven, R., Oner, M., Bayramoglu, B. The effectiveness of geriatric pneumonia severity index in predicting mortality. *International Journal of Gerontology*. 2021;15(1):73-77.

137. Yenibertiz, D., Aydin, M.S., Özyürek,B.A. What are the predictors of delirium for patients with lung cancer? *Turk Hijyen ve Deneysel Biyoloji Dergisi*. Published online 2021.

138. Yuksel H, Gursoy GT, Dirik EB, et al. Neurological manifestations of COVID-19 in confirmed and probable cases: A descriptive study from a large tertiary care center. *Journal of Clinical Neuroscience*. 2021;86:97. doi:10.1016/j.jocn.2021.01.002

139. Zerbit J, Detroit M, Chevret S, et al. Remdesivir for Patients Hospitalized with COVID-19 Severe Pneumonia: A National Cohort Study (Remdeco-19). *Journal of Clinical Medicine*. 2022;11(21):6545. doi:10.3390/jcm11216545

140. Zhang ZX, Yong Y, Tan WC, Shen L, Ng HS, Fong KY. Prognostic factors for mortality due to pneumonia among adults from different age groups in Singapore and mortality predictions based on PSI and CURB-65. *Singapore Med J*. 2018;59(4):190-198. doi:10.11622/smedj.2017079

141. Zukowska A, Kaczmarczyk M, Listewnik M, Zukowski M. The Association of Infection with Delirium in the Post-Operative Period after Elective CABG Surgery. *J Clin Med*. 2023;12(14):4736. doi:10.3390/jcm12144736

142. McGuinness LA, Higgins JPT. Risk-of-bias VISualization (robvis): An R package and Shiny web app for visualizing risk-of-bias assessments. *Research Synthesis Methods*. 2021;12(1):55-61. doi:10.1002/jrsm.1411

143. McGrath S, Zhao X, Ozturk O, Katzenschlager S, Steele R, Benedetti A. metamedian: An R package for meta-analyzing studies reporting medians. *Research Synthesis Methods*. 2024;15(2):332-346. doi:10.1002/jrsm.1686
